# Structure and Topology Dynamics of Hyper-Frequency Networks during Rest and Auditory Oddball Performance

**DOI:** 10.3389/fncom.2016.00108

**Published:** 2016-10-17

**Authors:** Viktor Müller, Dionysios Perdikis, Timo von Oertzen, Rita Sleimen-Malkoun, Viktor Jirsa, Ulman Lindenberger

**Affiliations:** ^1^Center for Lifespan Psychology, Max Planck Institute for Human DevelopmentBerlin, Germany; ^2^Institut National de la Santé et de la Recherche Médicale, Institut de Neurosciences des Systèmes UMR_S 1106, Aix-Marseille UniversitéMarseille, France; ^3^Department of Psychology, University of VirginiaCharlottesville, VA, USA; ^4^Centre National de la Recherche Scientifique, Institut des Sciences du Mouvement UMR 7287, Aix-Marseille UniversitéMarseille, France; ^5^Max Planck UCL Centre for Computational Psychiatry and Aging ResearchBerlin, Germany; ^6^Department of Political and Social Sciences, European University InstituteSan Domenico di Fiesole (FI), Italy

**Keywords:** functional connectivity, directional coupling, hyper-frequency network, network topology dynamics, graph-theoretical approach, resting state, auditory oddball performance

## Abstract

Resting-state and task-related recordings are characterized by oscillatory brain activity and widely distributed networks of synchronized oscillatory circuits. Electroencephalographic recordings (EEG) were used to assess network structure and network dynamics during resting state with eyes open and closed, and auditory oddball performance through phase synchronization between EEG channels. For this assessment, we constructed a hyper-frequency network (HFN) based on within- and cross-frequency coupling (WFC and CFC, respectively) at 10 oscillation frequencies ranging between 2 and 20 Hz. We found that CFC generally differentiates between task conditions better than WFC. CFC was the highest during resting state with eyes open. Using a graph-theoretical approach (GTA), we found that HFNs possess small-world network (SWN) topology with a slight tendency to random network characteristics. Moreover, analysis of the temporal fluctuations of HFNs revealed specific network topology dynamics (NTD), i.e., temporal changes of different graph-theoretical measures such as strength, clustering coefficient, characteristic path length (CPL), local, and global efficiency determined for HFNs at different time windows. The different topology metrics showed significant differences between conditions in the mean and standard deviation of these metrics both across time and nodes. In addition, using an artificial neural network approach, we found stimulus-related dynamics that varied across the different network topology metrics. We conclude that functional connectivity dynamics (FCD), or NTD, which was found using the HFN approach during rest and stimulus processing, reflects temporal and topological changes in the functional organization and reorganization of neuronal cell assemblies.

## Introduction

As noted by Prigogine and Stengers (1984, p. 77), “Nature speaks with thousand voices, and we have only begun to listen.” So does the human brain. The voices are neural oscillations, interacting with each other, transiently forming ensembles of varying synchrony across frequency bands, giving rise to an emergent whole, as voices do in a chorus. Interacting neurons in the brain dynamically self-organize into coherently oscillating structures or cell assemblies that are generated and activated by input from external or internal sources (Mayer-Kress, 1998). Separate cell assemblies communicate with each other to integrate the various information flows into a common network organization. One of the candidate mechanisms underlying integration and communication between cell assemblies is cross-frequency coupling, allowing accurate timing between different oscillatory rhythms (Jensen and Colgin, 2007; Jirsa and Müller, 2013), selective and dynamic control of distributed functional cell assemblies (cf. Canolty et al., 2010), and promotion of different dimensions of brain integration (Varela et al., 2001; Buzsáki and Draguhn, 2004; Buzsáki, 2006). Despite these general claims, surprisingly little is known about the mechanisms underlying complex interactions of spatially segregated cell assemblies. The present article aims to overcome the limitations of previous studies describing the oscillatory brain dynamics emerging at individual frequencies. Its purpose is to analyze and elucidate the network architecture and the *network dynamics based on within- and cross-frequency coupling* (WFC and CFC, respectively) in a common space, termed a *hyper-frequency network* (HFN), and how these change during rest and auditory oddball performance. HFN is defined here as a network that represents all interactions among frequencies and electrode sites (see below).

It is well known that temporally coherent brain activity can emerge in the absence of an explicit task (Ghosh et al., 2008; Deco et al., 2009, 2011). This so-called “resting state” activity and its underlying coupling dynamics can be captured at different scales (from a single cortical area to multiple cortical areas and whole brain dynamics) and frequencies using both neuroimaging techniques (fMRI and PET) and electroencephalographic (EEG) or magnetoencephalographic (MEG) recordings (Biswal et al., 1995; Greicius et al., 2003; Müller et al., 2003a,b; Damoiseaux et al., 2006; Deco et al., 2009; Venables et al., 2009). Computational studies (e.g., Ghosh et al., 2008; Deco et al., 2011) suggest that large-scale resting state networks are associated with coherent fluctuations that span a wide range of timescales, including those captured by imaging and EEG/MEG studies. Computational work also suggests that intrinsic noise and time delays via propagation along connecting fibers contribute to the dynamics of resting state networks (Ghosh et al., 2008; Deco et al., 2011).

There is evidence that CFC might play a crucial role in neuronal computation, communication, working memory, learning and other brain functions or processes (Canolty and Knight, 2010; Fell and Axmacher, 2011; Jirsa and Müller, 2013). Schack and Weiss (2005) showed that successful encoding of nouns was accompanied not only by increased phase synchronization within (measured by phase locking index) and between selected electrodes (measured by phase coherence) in the theta and the gamma frequency bands, but also by increased CFC or 1:6 phase synchronization at selected electrodes and between them. Isler et al. (2008) reported increased CFC for delta-theta (1:3) and delta-alpha (1:4) relationships in widespread fronto-central, right parietal, temporal, and occipital regions during auditory novelty oddball task. In a MEG study (Palva et al., 2005), enhanced phase-to-phase CFC was found among alpha, beta, and gamma frequency oscillations during continuous mental arithmetic tasks. Interestingly, in full-term newborns, CFC was reported between two delta rhythms (1–1.5 and 3.5–4.5 Hz) characterizing specific oscillatory interactions during the typical trace alternant burst activity (Wacker et al., 2010). Thus, functional connectivity within and between different oscillation frequencies and brain regions reflects and supports major cognitive functions, neural communication, and plasticity.

In a previous study, Müller and Lindenberger (2012) demonstrated that methods and models derived from nonlinear dynamics are suitable tools for describing resting state networks and their changes during task performance. Specifically, the authors showed that nonlinear coupling was higher during resting state with eyes closed than with eyes open, whereas the reverse pattern was found for dynamic complexity. During stimulus processing, there was a significant drop in complexity and a rise in nonlinear coupling. Using another complexity measure (MSE, multi-scale entropy) for comparison of resting state and oddball performance in young and older adults, Sleimen-Malkoun et al. (2015) found that the EEG of the attended oddball task, especially in young adults, was less complex at shorter time scales but more complex at longer time scales. Furthermore, Müller et al. (2009) found that oscillatory brain activity and the corresponding phase synchronization dynamics are modulated during stimulus processing and task performance. Finally, Jirsa and Müller (2013) recently showed that CFC measures covering the interaction between different frequencies add another dimension to the understanding of complex neural dynamics of the frequency-specific neuronal networks. The authors suggested that CFC may allow accurate timing between different oscillatory rhythms, thereby facilitating communication between different cell assemblies. Specifically, they found that delta and alpha frequency interactions play a crucial role in resting state networks (Jirsa and Müller, 2013). Recently, Aru et al. (2015) criticized physiological interpretations of CFC, in particular phase-to-amplitude CFC. In simulated data, Jirsa and Müller (2013) provided support thereof demonstrating that phase-to-amplitude CFC can indeed show spurious results via smearing of coupling across stimulation frequencies, whereas phase-to-phase CFC shows high precision in detecting stimulation frequencies, even in presence of noise. In the present study, we go a step further by using WFC and CFC for the construction of a complex network that includes all interactions within and between the frequencies across space and time. The advantage of this approach has been recently shown in an inter-brain study on kissing (Müller and Lindenberger, 2014).

A growing body of evidence from electromagnetic and neuroimaging studies suggests that functional connectivity (FC) is non-stationary and that fluctuations of FC-based networks produce specific functional connectivity dynamics (FCD) that can be understood as a manifestation of the self-organized activity of cortical or neural networks (Chavez et al., 2010; Betzel et al., 2012; Chu et al., 2012; Hutchison et al., 2013; Leonardi et al., 2013; Messé et al., 2014; Hansen et al., 2015; Yu et al., 2015). In these networks in resting state, noise-driven fluctuations far from equilibrium provide a rich repertoire of characteristic system trajectories (Hansen et al., 2015).

Here, we present EEG data obtained from 58 electrodes at rest with eyes closed (REC) and open (REO), and during an auditory oddball task under attended (AOT) and unattended (UOT) conditions. To determine WFC and CFC between different electrodes, we use phase synchronization algorithms described in previous studies that measure directed and undirected phase-to-phase coupling (Müller and Lindenberger, 2011, 2014; Müller et al., 2013). These coupling measures were used to construct a connectivity matrix or a graph representing the network properties. In contrast to earlier approaches, where different brain sites (different electrodes in the case of the EEG) were defined as nodes in such a graph, we defined nodes as a combination of site and frequency. This means that each electrode is represented by 10 different nodes corresponding to 10 frequencies of interest (FOIs) in the frequency range between 2 and 20 Hz (in steps of 2 Hz) that communicate with other nodes at the same or different frequencies. The advantages of a network architecture allowing for WFC and CFC are: (1) not only connections between, but also within brain areas can be captured, and (2) different brain areas can communicate with each other at multiple frequencies (Müller and Lindenberger, 2014). There were 580 nodes altogether (58 electrodes × 10 frequency bins = 580 nodes) in the common network. In these so-called HFNs, we computed different graph-theoretical approach (GTA) measures and investigated their temporal changes in time, i.e., network topology dynamics (NTD). NTD was investigated by using six graph-theoretical measures such as in- and out-strengths, clustering coefficient, characteristic path length (CPL), local, and global efficiency. The in- and out-strengths indicate incoming and outgoing connections of the network nodes, respectively, and are measures of network connectivity. The clustering coefficient (*CC*) measures cliquishness of a typical neighborhood and is a measure of network segregation, whereas the *CPL* measures a typical separation between two nodes and shows the degree of network integration, with a short *CPL* indicating higher network integration (Watts and Strogatz, 1998). Like *CC*, local efficiency (*E*_*loc*_) is a measure of the segregation of a network, indicating efficiency of information transfer in the immediate neighborhood of each node and showing how fault-tolerant the system is (Latora and Marchiori, 2001). Similar to *CPL,* global efficiency (*E*_*glob*_) is a measure of the integration of a network, but whereas *CPL* is primarily influenced by long paths, *E*_*glob*_ is primarily influenced by short ones (Latora and Marchiori, 2001). From an organizational point of view, networks indicated by high *CC* and shorter *CPL* have been described as small-world networks (SWN) that are also characterized by a high local and global efficiency of parallel information transfer (Achard and Bullmore, 2007).

In addition, we introduce an approach to reveal stimulus-related NTD here by using feed-forward neural network (FNN) classification algorithm for the artificial neural network to learn to identify clusters in NTD data that are related to the stimulus structure and then to test the performance of this network by using training and testing sets, respectively (Alpaydın, 2010). We investigated the stimulus-related NTD for the six GTA measures mentioned above under the two oddball-task conditions (UOT and AOT) separately for 10 different oscillation frequencies combining 58 electrodes within the HFN.

## Methods

### Participants

All participants were volunteers recruited via announcements at Saarland University and were provided with a description of the study to obtain written informed consent. All participants were paid 7.50 Euro per hour to take part in the study. They were all right-handed, had no reported history of head injuries or neurological disorders, and were not on medication. The sample consisted of 31 participants (mean age = 22.6, SD = 1.6, age range = 18.8–25.1 years, 14 females). The study was approved by the ethics committee of Saarland University and was thus performed in accordance with the ethical standards laid down in the 1964 Declaration of Helsinki.

### Procedure

The EEG measurement began with a 3-min relaxation phase (1.5 min with eyes closed and 1.5 min with eyes open). Instructions for the resting states were given on the computer display and were presented as follows: “A cross will be shown in the middle of the screen for a minute and a half. Please focus on the cross and relax” (for the REO condition) and “Keep your eyes closed for a minute and a half and relax” (for the REC condition). The rest phases were then followed by the auditory oddball task. During the recording, the subjects sat in a chair in a relaxed position in an electrically shielded room. During the oddball task, which was carried out with eyes closed, the participants heard two different types of tone pips: a 1000 Hz tone played frequently to form the standard stimulus and a 800 Hz tone played only intermittently to form the deviant stimulus. The standard and deviant stimuli were presented binaurally (with a probability of 0.8 and 0.2 for the standard and deviant stimuli, respectively) through headphones (Sony DJ MDR-V300) at 70 dB SPL for a duration of 70 ms (including a 10-ms rise and fall period). The stimuli were generated using the Audacity 1.2.4 software. The inter-stimulus interval (ISI) was uniformly chosen at random between 1200 and 1500 ms. Two different experimental conditions were used: passive listening (UOT) and active counting (AOT). For the first condition, the subjects were simply asked to listen to the tone pips without any response, whereas, for the second condition, the subjects were asked to listen to the stimuli and count the number of deviant tones. They only had to report back the number of tones counted once the session was complete. Each experimental condition contained 152 standard tones and 38 deviant tones presented in a pseudo-random order fixed for all participants. The conditions were always presented in the same order, with the passive listening condition followed by the active counting condition in order to facilitate the interpretation of between-person differences.

### EEG recordings and analyses

The electroencephalogram (EEG) was recorded from 58 Ag/AgCl electrodes using an elastic cap (Electrocap International) with a sampling rate of 500 Hz in a frequency band ranging between 0.5 and 100 Hz. The left mastoid was used as a reference and the right mastoid was recorded as an active channel. The data were also re-referenced off-line to an average of the left and right mastoids for further analysis. The electrodes were placed according to the international 10–10 system. The vertical and horizontal electrooculograms (EOG) were recorded for control of eye blinks and eye movements. Signals were digitally filtered off-line (Butterworth zero phase filters 1–100 Hz, slope 12 dB/octave; notch filter 50 Hz). Eye movement correction was accomplished by independent component analysis (Vigário, 1997) using BrainVision Analyzer (Brain Products, Gilching, Germany). Thereafter, artifacts from head and body movements were rejected by visual inspection. Finally, data were downsampled to a sampling rate of 250 Hz, segmented in artifact-free 10-s segments (i.e., comprising *N*_*t*_ = 2500 data points each), and normalized within segments before further analysis.

### Phase coupling measures

**Figure 1:**
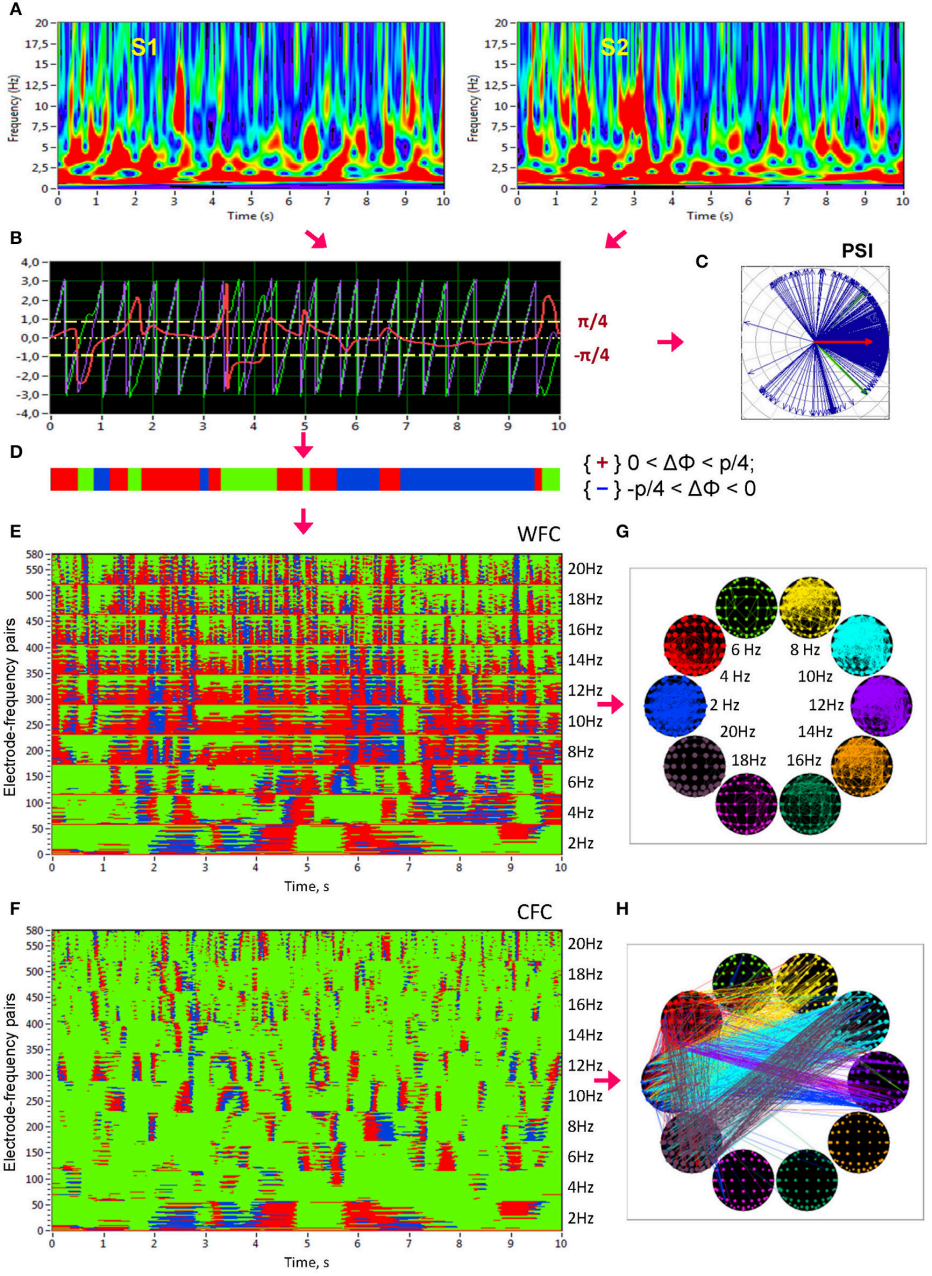
**Schematic presentation of phase synchronization assessment. (A)** Complex Morlet wavelet transformation of signals from two channels **(A,B)** in the time-frequency domain. **(B)** Time course of instantaneous phases from these two channels and their phase difference (**A**, violet curve; **B**, green curve; **A,B**, red curve). **(C)** The phase difference is depicted in form of the vectors in complex space, where the blue arrows reflect single phase angles and the red arrow represents the mean vector of the angular dispersions; its length displays the *PSI* measure. **(D)** Coding of the phase difference of two signals at a given frequency (−π/4 < A–B < 0: blue stripes; 0 < A–B < +π/4: red stripes; A–B < −π/4 or S1–S2 > +π/4: green stripes = non-synchronization). Note that these phase differences, coded with +1 (red), 0 (green), or −1 (blue) at each time point, are used to calculate the four synchronization measures (i.e., *PCI, NCI, ACI,* and *ICI*) described in the Methods. **(E)** Pair-wise synchronization pattern of WFC from one channel (Fpz) to all other channels at 10 FOIs (58 × 10 = 580 lines). Each line represents the coded phase difference as described above. **(F)** Pair-wise synchronization pattern of CFC from one channel (Fpz) oscillating at the frequency of 2 Hz to all other channels oscillating at 10 different frequencies (58 × 10 = 580 lines). **(G)** Brain maps of WFC indicated by ICI (Integrative Coupling Index) above the threshold (ICI > 0.45). **(H)** Brain maps of CFC indicated by ICI above the threshold (ICI > 0.40). These thresholds in **(G,H)** were chosen to ensure the visualization of the maps. Only the strongest connections are depicted

To investigate phase coupling in a directed and frequency-resolved manner (cf. Müller et al., 2013), we applied an analytic or complex-valued Morlet wavelet transform to compute the instantaneous phase in the frequency range from 0 to 20 Hz in 0.125-Hz steps (see Figure 1A). The complex mother Morlet wavelet, also called Gabor wavelet, has a Gaussian shape around its central frequency *f*:

(1) $$\begin{array}{l} w\left(t,f\right)={\left(\sigma^{2}\pi \right)}^{-1/4}e^{\left(\left(-t^{2}/2\sigma^{2}\right)\;+\;3/2\pi jft\right)},j=\sqrt{-1} \end{array}$$

in which σ is the standard deviation of the Gaussian envelope of the mother wavelet. The wavelet coefficients were calculated with a time step of 5, leading to a time resolution of 20 ms and frequency resolution of 0.125 Hz. In order to identify the phase relations within and between any two channels or frequencies, the instantaneous phase difference was then computed from the wavelet coefficients for all possible electrode and frequency pairs (Figure 1B). On the basis of instantaneous phases for two signals (*X* and *Y*) given as: Φ_*X*_(*f*_*m*_*,t*) = arg[ϕ_*X*_(*f*_*m*_*,t*)] and Φ_*Y*_(*f*_*n*_*,t*) = arg[ϕ_*Y*_(*f*_*n*_*,t*)], correspondingly, with ϕ_*X*_ and ϕ_*Y*_ being complex numbers, the *n:m* phase synchronization between two oscillations at the frequencies *f*_*m*_ and *f*_*n*_ was determined. The generalized phase difference (ΔΦ) according to *n*· *f*_*m*_ = *m*· *f*_*n*_ was calculated by:

(2) $$\begin{array}{l} \Delta \Phi \left(f_{m},f_{n},t\right)=n\cdot \Phi \left(f_{m},t\right)-m\cdot \Phi \left(f_{n},t\right),mod2\pi \end{array}$$

In the case of WFC with *f*_*m*_ = *f*_*n*_, the phase difference ΔΦ is calculated in the same way by setting *m* = *n* = 1.

The *n:m* phase synchronization index (*PSI*) was then defined by:

(3) $$PSI\left(f_{m},f_{n}\right)=|\langle e^{j\cdot \Delta \Phi \left(f_{m},f_{n},t\right)}\rangle |\;,j=\sqrt{-1}$$

where < ∙> denotes the averaging across *time*. The *PSI* is similar to phase coherence, with the difference that the *PSI* measures phase stability or phase invariance *across time* within a trial or time series (Figure 1C).

In addition to *PSI*, which is independent of the phase angle in the sense that *PSI* can be high at different phase angle differences (e.g., signals oscillating in anti-phase would also obtain a high *PSI* value), we calculated further synchronization indexes reflecting in-phase synchronization between two electrodes, that is, the extent to which the angle of phase differences approximates 0. Given the estimates of the phase difference between pairs of signals, it is possible to determine for how long the phase difference remains stable in defined phase angle boundaries by counting the number of points that are phase-locked in a defined time window. We adapted and slightly modified the procedure described in Kitzbichler et al. (2009) in that we divided the range between −π/4 and +π/4 into two ranges and distinguished between positive and negative deviations from phase zero. As shown in Figure 1D, we marked negative deviations in the range between −π/4 and 0 in blue (coded with “−1”) and positive deviations in the range between 0 and +π/4 in red (coded with “+1”). Phase difference values beyond these ranges were marked green (coded with “0”) and represent non-synchronization. In the case of two channels, A and B, a blue stripe in the diagram would mean that the phase of channel B precedes that of channel A, and a red stripe would mean that the phase of channel A precedes that of channel B.

We then counted the number of data points that are phase-locked separately in each of these two ranges. Before counting, successive points in the defined range (between −π/4 and +π/4) with a time interval shorter than a period of the corresponding oscillation at the given frequency (*T*_*i*_ = 1/*f*_*i*_) were discarded from the analysis. This cleaning procedure effectively eliminated instances of accidental synchronization. Synchronization patterns of WFC (580 lines or electrode pairs of channel A to all other channels within the 10 frequencies) and CFC (580 lines or electrode pairs of channel A at FOI = 2 Hz to all other channels at the 10 different frequencies) after the cleaning procedure are presented in Figures 1E,F, respectively. On the basis of this counting, we obtained several synchronization indices: (1) the Positive Coupling Index, *PCI*, or the relative number of phase-locked points in the positive range (between 0 and +π/4); (2) the Negative Coupling Index, *NCI*, or the relative number of phase-locked points in the negative range (between −π/4 and 0); (3) the Absolute Coupling Index, *ACI*, or the relative number of phase-locked points in the positive and negative range (i.e., between −π/4 and +π/4) indicating in-phase synchronization; (4) the Integrative Coupling Index, *ICI*, calculated by the formula (Müller and Lindenberger, 2011):

(4) $$\begin{array}{l} ICI=\frac{PCI\;+\;ACI}{2\cdot ACI}\cdot \sqrt{PCI} \end{array}$$

All these coupling measures are related to all measurement points in the window and range from 0 and 1. *PSI* and *ACI* are symmetrical measures (i.e., *PSI*_AB_ = *PSI*_BA_ and *ACI*_AB_ = *ACI*_BA_) and have similar properties when synchronization is in phase, whereas the *ICI* is asymmetric (*ICI*_AB_ ≠ *ICI*_BA_), indicating the relative extent of positive phase synchronization. The ICI is equal to 1 when all points are phase-locked and are in the positive range; if all phase-locked points lie in the negative range, the term $\frac{PCI\;+\;ACI}{2\cdot ACI}$ will approach 0.5 but through multiplication with $\sqrt{PCI}$, it will approach 0. The *ICI* approximately equals 0.5 when half of the phase-locked points lie in the positive range, and the other half of phase-locked points lie in the negative range. Moreover, by using the framework of “The Virtual Brain” (TVB, www.thevirtualbrain.org), simulation results in our previous study (Müller et al., 2013) showed that all three measures (*PSI, ACI*, and *ICI*,) capture the intended coupling properties. Particularly *ICI* shows a peak in the middle of the considered positive interval (between 0 and π/4) at π/8. In this article, we only report results on the *ICI* measure, which is the most informative due to its directionality. Other synchronization measures (e.g., *PSI* and *ACI*) were also computed but will not be reported here for space reasons. *ICI*-based brain maps comprising all electrode pairs of WFC (with *ICI* > 0.55) and CFC (with *ICI* > 0.32) are presented in Figures 1G,H, respectively. Note that for visualization reasons, only the strongest connections are displayed.

In order to investigate the dynamic changes in phase synchronization and network topology (see below), we calculated phase coupling using a moving time window of 2000 ms width and 100 ms time delay. Overall, within a segment of 10 s duration, coupling measures across 81 time widows were collected by this shifting procedure.

### Graph-theoretical approach (GTA) and network metrics

#### Network construction

**Figure 2:**
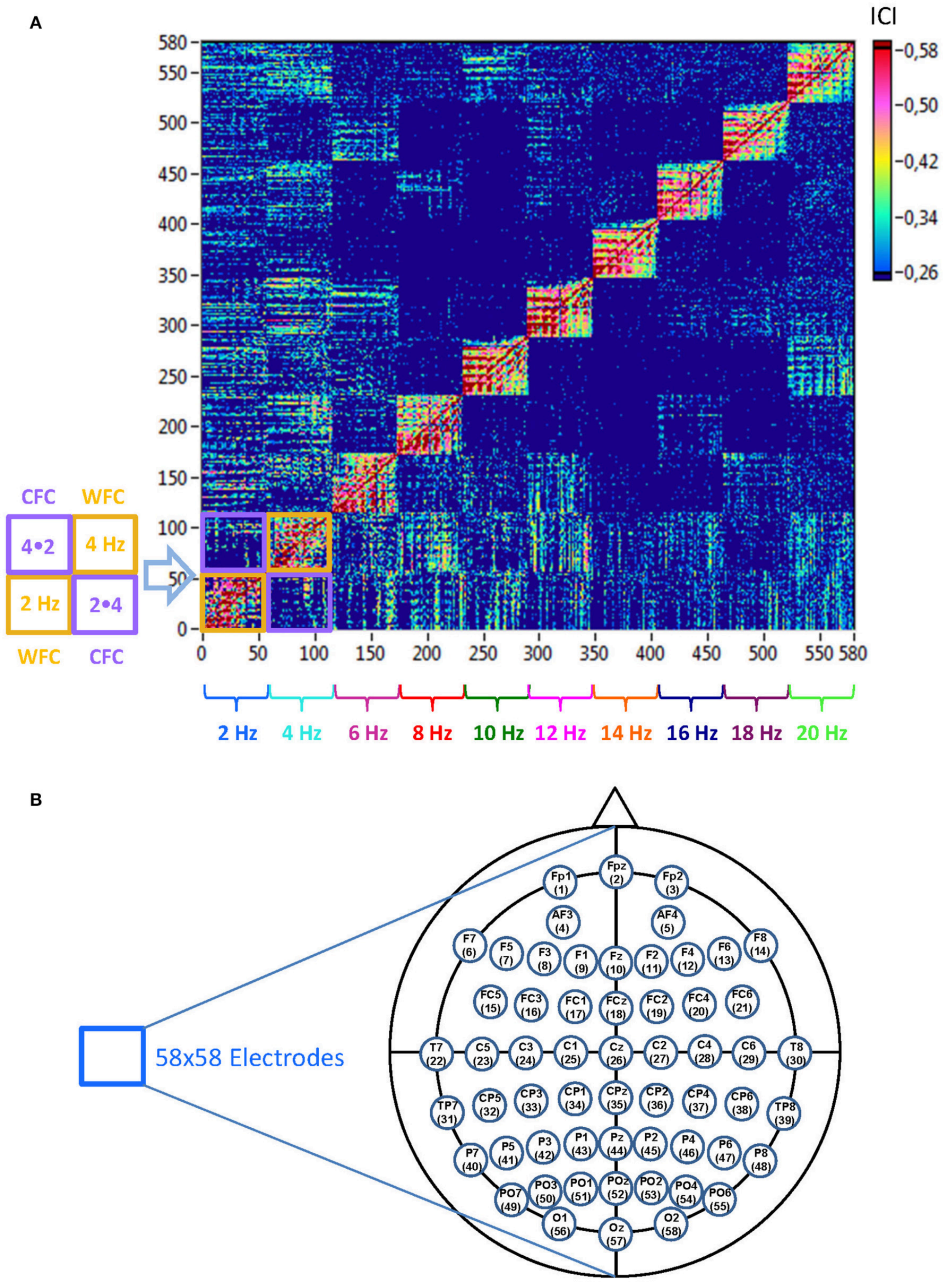
**Schematic presentation of a Hyper-Frequency Network (HFN). (A)** Network structure: HFN consists of 580 nodes representing 58 electrodes oscillating at 10 different frequencies (2, 4, 6, …, 20 Hz). Thus, each node is a combination of spatial representation (electrode location) and the oscillation frequency. HFN constructed in this way integrate WFC and CFC, whereby WFC nodes are placed along the diagonal. To the left of the network examples of WFC (2 and 4 Hz) and CFC (2–4 Hz and 4–2 Hz) blocks with 58 electrodes each are presented schematically. **(B)** Electrodes map with electrode positions and the order (indicated by number), in which they appear in the network.

The coupling measures (determined in the frequency range from 2 to 20 Hz in 2-Hz steps) were used to construct a connectivity matrix or a graph representing the network properties, where each node is defined as a combination of electrode location and oscillation frequency. This resulted in a common network with 580 nodes (58 electrodes × 10 frequency bins). The structure of such a graph is represented in Figure 2 and is considered as a directed weighted graph in further analyses.

To investigate the network topology of the HFN, we also constructed regular (lattice) and random networks with the same number of nodes and mean degree as our real networks. For this purpose, we randomized the edges in the respective real network to achieve a random network with the same number of nodes and edges. Lattice networks were configured like random networks, but in addition the edges were redistributed after an initial random permutation such that they lay close to the main diagonal with increasing order of their weights. To do this, each column in the adjacency matrix was split into two parts at the diagonal element. All edges in these two parts were sorted by order (with the largest closest to the diagonal element), and then merged again into one column. Lattice networks reconstructed in such a way have the same number of nodes and edges as the initial real network but are characterized by ring or lattice topology incorporating nearest-neighbor connectivity (Sporns et al., 2007). These network reconstructions for random and regular networks were carried out 10 times for each individual network. Average network topology was then determined for these repeated reconstructions.

#### Threshold determination

In general, the choice of a threshold plays an important and non-trivial role in network construction, but is necessarily always arbitrary. At least two issues appear important in this study: (1) the connectivity measures should not occur by chance, and (2) the networks changing in time should have the same threshold, which correspond to a high sparsity level. To determine the network properties across the different time windows, we set the connectivity threshold to 0.26, which was always higher than the significance level determined by the surrogate data procedure (see below). At this threshold, the cost level of the networks (ratio of the number of actual connections divided by the maximum possible number of connections in the network) was ~20%, corresponding to high sparsity of the resulting networks and allowing more accurate examination of the network topology.

Surrogate data were created in two ways: (1) by random permutations of the original time series, and (2) by phase permutation of the time series. The latter surrogate data procedure involved: (a) computing the amplitude and phase spectrum of a real signal using a Fourier transformation; (b) phase shuffling, whereby the phase values of the original spectrum are used in random order and the sorted values of the surrogate sequence are replaced by the corresponding sorted values of the reference sequence; and (c) inverse Fourier transformation back to the time domain. In this way, the real and the surrogate data retain the same power spectrum but a different time course. Thereafter, we applied a bootstrapping procedure with 1000 resamples of the coupling measures resulting from the surrogate data sets and determined the significance level (*p* < 0.0001) as the bootstrapping mean plus the confidence interval. The chosen threshold of 0.26 was always higher than the determined significance level in both surrogate data procedures and corresponded to a relatively high sparsity level, i.e., it matched both of our criteria (see above).

#### Degrees and strengths

As *ICI* is a directed measure, we obtained the node in- and out-degrees in the network, the in-degree being the sum of all incoming connections of the node *i* $k_{i}^{in}=\underset{j\in N}{\sum }a_{ji}$, and the out-degree being the sum of all outgoing connections $k_{i}^{out}=\underset{j\in N}{\sum }a_{ij}$. To calculate strengths, we then replaced the sum of links by the sum of weights, $k_{i}^{w}=\underset{j\in N}{\sum }w_{ij}$, and calculated the in- and out-strengths, respectively. Thus, the strength can be regarded as the weighted degree (Rubinov and Sporns, 2010).

#### Clustering coefficient and characteristic path length

If the nearest neighbors of a node are also directly connected to each other, they form a *cluster*. For an individual node, the *CC* is defined as the proportion of the existing number of connections to the total number of possible connections. In the case of a weighted directed graph the mean *CC* is calculated by the formula (Fagiolo, 2007):

(5) $$\begin{array}{rcl} CC^{wd} & = & \frac{1}{n}\underset{i\in N}{\sum }CC_{i}^{wd}\\ =\frac{1}{n}\underset{i\in N}{\sum }\frac{t_{i}^{wd}}{\left(k_{i}^{out}+k_{i}^{in}\right)\left(k_{i}^{out}+k_{i}^{in}-1\right)-2\underset{j\in N}{\sum }a_{ij}a_{ji}} \end{array}$$

with $t_{i}^{wd}=\frac{1}{2}\underset{j,h\in N}{\sum }{\left[\left(w_{ij}^{1/3}w_{ih}^{1/3}w_{jh}^{1/3}\right)+\left(w_{ji}^{1/3}w_{hi}^{1/3}w_{hj}^{1/3}\right)\right]}^{3}$ being the number of weighted directed triangles around a node *i*.

Another important measure is the *CPL*. In an unweighted graph, the shortest path length or distance *d*_*i, j*_ between two nodes *i* and *j* is the minimal number of edges that have to be passed to go from *i* to *j*. This is also called the geodesic path between the nodes *i* and *j*. The *CLP* of a graph is the mean of the path lengths between all possible pairs of vertices (Watts and Strogatz, 1998):

(6) $$\begin{array}{l} CPL=\frac{1}{n}\underset{i\in N}{\sum }L_{i}=\frac{1}{n}\underset{i\in N}{\sum }\frac{\Sigma_{j\in N,j\neq i}\;d_{ij}}{n-1} \end{array}$$

where *L*_*i*_ = *CPL*_*i*_ is the average distance or average shortest path length between node *i* and all other nodes. As our networks are directed weighted graphs, the weight and direction of the links are used in this calculation.

#### Local and global efficiency

*E*_*loc*_ is similar to the *CC* and is calculated as the harmonic mean of neighbor-neighbor distances (Latora and Marchiori, 2001):

(7) $$E_{loc}^{w}=\frac{1}{n}\underset{i\in N}{\sum^{\text{​}}}\frac{\sum j,h\in N,j\neq i(w_{ij}w_{ih}{\left({\left[d_{jh}^{w}\left(N_{i}\right)\right]}^{-1}\right)}^{1/3}}{k_{i}(k_{i}-1)}$$

Like *CC, E*_*loc*_ is a measure of the segregation of a network, indicating efficiency of information transfer in the immediate neighborhood of each node and showing how fault-tolerant the system is.

*E*_*glob*_ is defined as the average inverse shortest path length and is calculated by the formula (Latora and Marchiori, 2001):

(8) $$\begin{array}{l} E_{glob}^{w}=\frac{1}{n}\underset{i\in N}{\sum }\frac{\underset{j\in N,j\neq i}{\sum }{\left(d_{ij}^{w}\right)}^{-1}}{n-1} \end{array}$$

Like *CPL, E*_*glob*_ is a measure of the integration of a network, but whereas *CPL* is primarily influenced by long paths, *E*_*glob*_ is primarily influenced by short ones. Calculating *E*_*glob*_ is advantageous over distance in disconnected networks: The efficiency between disconnected pairs of nodes is set to zero (the inverse of infinity).

Since we were interested in nodal network characteristics for our further analyses, we determined all the GTA measures described above for each node separately.

#### Small-worldness

To investigate the *small-world* (SW) properties of a network it has become common to compare its clustering coefficient and CPL to those of regular lattices and random graphs. At least two specific properties of small-world network (SWN) related to control networks (random and lattice) are significant: (1) The *CC* of the SWN (*CC*_*SWN*_) is much higher than that of random networks (*CC*_*SWN*_ >> *CC*_*rand*_), but the CPL of the SWN (*CPL*_*SWN*_) is only slightly higher than that of the random network (*CPL*_*SWN*_ ≥ *CPL*_*rand*_), and (2) the *CC* of the SWN is lower than that of lattice networks (*CC*_*SWN*_ ≤ *CC*_*latt*_), but the *CPL* of the SWN is much lower than that of the lattice network (*CPL*_*SWN*_ < < *CPL*_*latt*_). Specific quantitative SW metrics were developed in addition to these main graph metrics. Foremost, the so-called SW coefficient σ, is related to the main metrics of a random graph (*CC*_*rand*_ and *CPL*_*rand*_) and is determined on the basis of two ratios γ = *CC*/*CC*_*rand*_ and λ = *CPL*/*CPL*_*rand*_ (Humphries et al., 2006):

(9) $$\begin{array}{l} \sigma =\frac{\gamma }{\lambda }=\frac{CC/CC_{rand}}{CPL/CPL_{rand}} \end{array}$$

The SW coefficient σ has been used in numerous networks showing SW properties and has been found to be >1 in the SWN.

The second SW metric was defined by comparing the *CC* of the network of interest to that of an equivalent lattice network and comparing the *CPL* of the network to that of an equivalent random network (Telesford et al., 2011):

(10) $$\begin{array}{l} \omega =\frac{CPL_{rand}}{CPL}-\frac{CC}{CC_{latt}} \end{array}$$

This metric normally ranges between −1 and +1 and is close to zero for SWN (*CPL*_*SWN*_ ~ *CPL*_*rand*_ and *CC*_*SWN*_ ~ *CC*_*latt*_). In addition, positive values of ω indicate a graph with more random characteristics (*CPL*_*SWN*_ ~ *CPL*_*rand*_ and *CC*_*SWN*_ < < *CC*_*latt*_), while negative values indicate a graph with more regular (lattice-like) characteristics (*CPL*_*SWN*_ >> *CPL*_*rand*_ and *CC*_*SWN*_ ~ *CC*_*latt*_). The clear advantage of the ω metric as compared to σ is the possibility to define the extent to which the network of interest is like its lattice or random equivalents (Telesford et al., 2011).

In addition, we reported σ*E* and ω*E* metrics here, which were determined on the basis of *E*_*loc*_ and *E*_*glob*_ instead of *CC* and *CPL*, using the same logic. The coefficient σ*E* was calculated by the formula:

(11) $$\begin{array}{l} \sigma E=\frac{E_{loc}/{\left(E_{loc}\right)}_{rand}}{{\left(E_{glob}\right)}_{rand}/E_{glob}}, \end{array}$$

and coefficient ω*E* was determined as:

(12) $$\begin{array}{l} \omega E=\frac{E_{glob}}{{\left(E_{glob}\right)}_{rand}}-\frac{E_{loc}}{{\left(E_{loc}\right)}_{latt}}. \end{array}$$

#### Network topology dynamics

**Figure 3:**
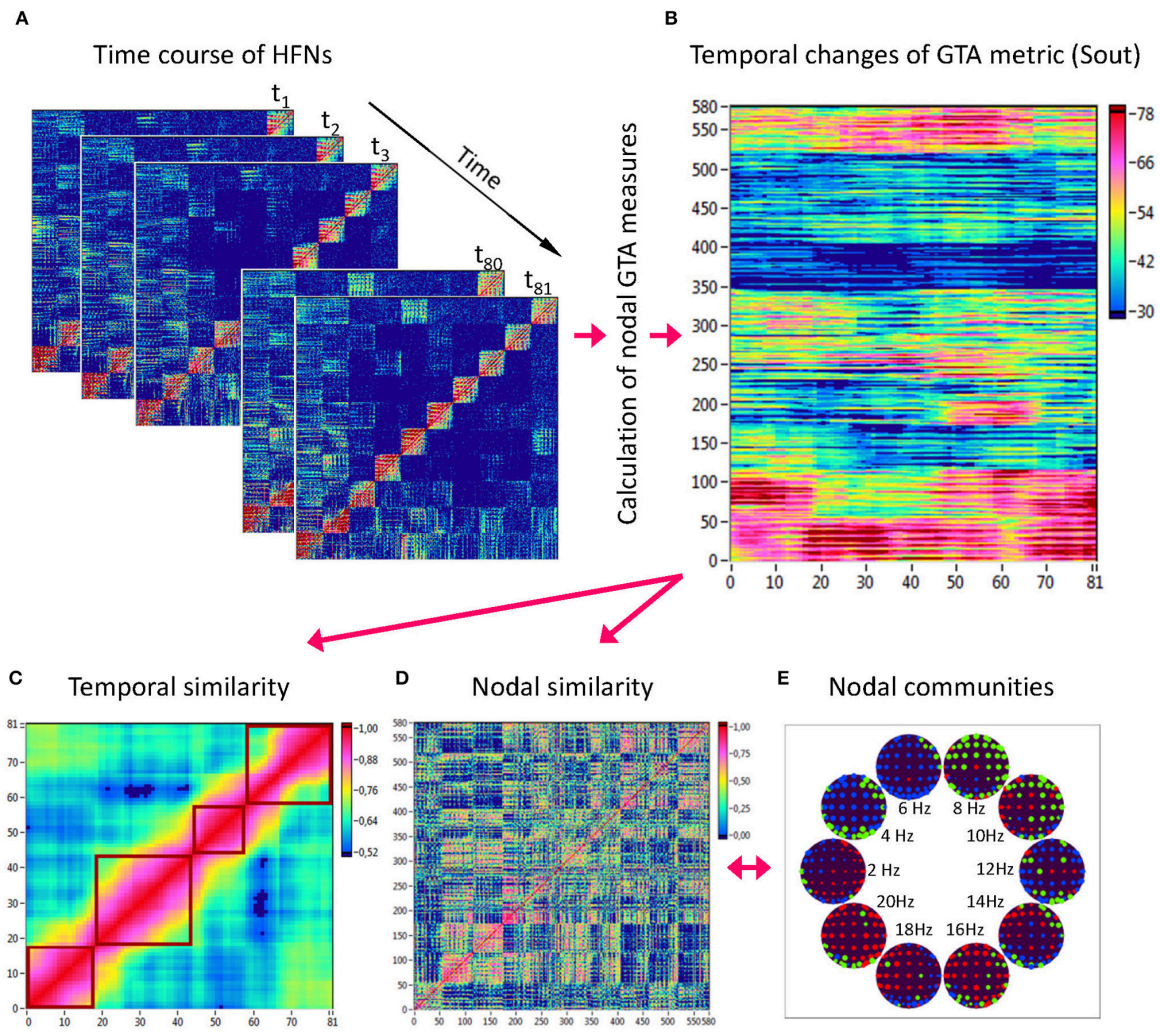
**Determination of HFN dynamics with temporal and nodal similarity. (A)** Time course of HFNs calculated in the 10-s segment using a moving time window of 2 s and a time delay of 100 ms (81 time windows in total). **(B)** After the calculation of different GTA metrics for HFN in each time window, a time window × nodes matrix (81 × 580) was constructed for each GTA metric (e.g., *Sout*). **(C)** The temporal similarity matrix was built by calculation of Pearson's product correlation among the consecutive vertical lines in the previous matrix (each line represents 580 nodes of a GTA metric in the corresponding time window). Using modularity analysis, dynamic states were determined in the temporal similarity matrix indicating a similar time course of a GTA metric of HFN. Different dynamic states are presented as quadrants along the diagonal of the temporal similarity matrix. **(D)** The nodal similarity matrix was built by calculation of Pearson's product correlation among the consecutive horizontal lines in previous 81 × 580 matrix (each line represents the time course of a single node of a GTA metric). **(E)** Using Louvain modularity analysis, nodal communities were determined in the nodal similarity matrix that comprise nodes with a similar time course. Nodal communities are color coded in brain maps of 10 different oscillation frequencies.

Network topology given by the GTA measures specified above changes across time. To capture the spatiotemporal NTD, we calculated the GTA metrics specified above for each time window and each HFN node (Figure 3A), then built a nodes × time windows matrix (580 × 81) for each GTA metric (Figure 3B). First, we gathered means and standard deviations across time and nodes to estimate the impact of different nodes and its changes in time as well as variability of GTA metrics in time and space (electrode positions and oscillation frequencies). Thereafter, we calculated *temporal network similarity*, that is, the correlations among consecutive time windows (Figure 3C), and spatial or *nodal network similarity*, that is, the correlations among consecutive nodes (Figure 3D). For both similarity measures, similarity was determined by Pearson's product correlation. We used modularity analysis for (1) identification of sequences of *coherent states* (81 × 81 correlation matrix indicating temporal network similarity), and (2) identification of node communities remaining stable or similar across time (580 × 580 correlation matrix indicating spatial/nodal network similarity). Compared to the k-means clustering analysis, which was used for investigation of FCD (cf. Hansen et al., 2015), modularity analysis uses optimization algorithms to detect optimized community structures and do not require pre-specification of the number of clusters or modules (Newman, 2004). For this calculation, the modularity optimization method for weighted networks as implemented in the Brain Connectivity Toolbox (Rubinov and Sporns, 2010) was used. The optimal community structure is a subdivision of the network or graph into non-overlapping groups of nodes in a way that maximizes the number of within-module edges, and minimizes the number of between-module edges. The modularity (*Q*) is a statistic that quantifies the degree to which the network may be subdivided into such clearly delineated groups or modules. For weighted networks, it is given by the formula (Newman, 2006):

(13) $$\begin{array}{l} Q^{w}=\frac{1}{l^{w}}\underset{j\in N}{\sum }\left[w_{ij}-\frac{k_{i}^{w}k_{j}^{w}}{l^{w}}\right]\cdot \delta_{m_{i}m_{j},} \end{array}$$

where *l*^*w*^ is the total number of edges in the network, N is the total number of nodes in the network, *w*_*ij*_ are connection weights, $k_{i}^{w}$ and $k_{j}^{w}$ are weighted degrees or strengths of the nodes, and δ_*m*_*i*_, *m*_*j*__ is the Kronecker delta, where δ_*m*_*i*_, *m*_*j*__ = 1 if *m*_*i*_ = *m*_*j*_, and 0 otherwise. High modularity values indicate strong separation of the nodes into modules, while Q will be zero if nodes are placed into modules at random or if all nodes are in the same module or cluster. Since spatial/nodal network similarity contains negative values besides the positive, we used the Louvain modularity algorithm provided for sign (positive and negative) correlation values in this case (Blondel et al., 2008). To test the modularity of the empirically observed networks, we compared them to the modularity distribution (*N* = 100) of random networks, that is, to simulated networks with the same number of nodes and edges as the original network (Bassett et al., 2010).

Coherent states identified by using modularity analysis are depicted in Figure 3C as quadrants along the diagonal of the temporal similarity matrix; they indicate phases with high temporal similarity. Nodes showing similar NTD are combined in so-called *nodal communities* indicated by color in Figure 3E. On the basis of these modularity analyses, we determined the number of dynamic states (modules) and the number of nodal communities, the minimal and the maximal duration of dynamic states as well as the minimal and the maximal size of nodal communities for all the GTA measures under the four task conditions.

#### Stimulus-related NTD

**Figure 4:**
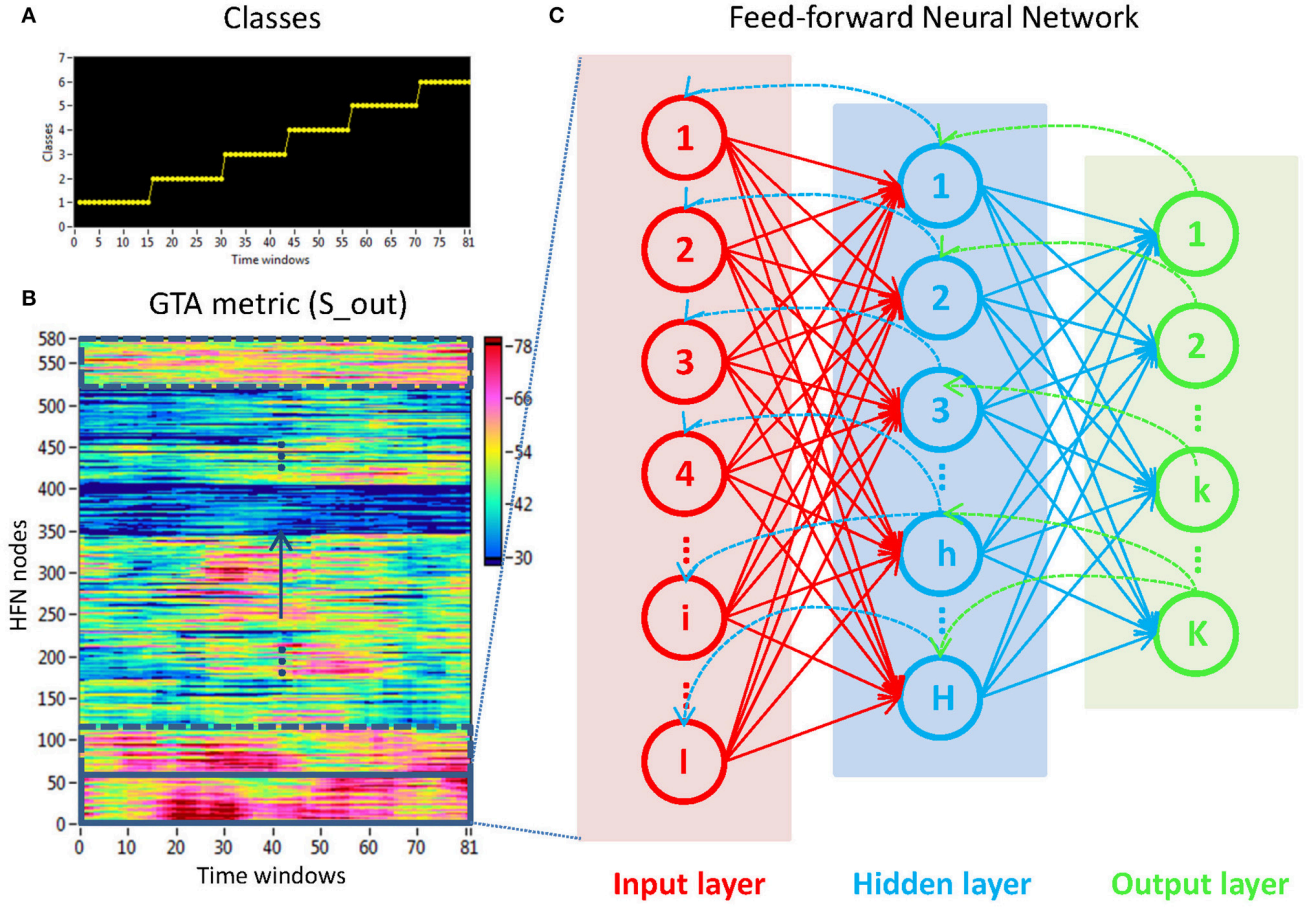
**Determination of stimuli-related NTD using the FNN approach. (A)** Stimulation structure with different classes representing certain ISIs. **(B)** Time windows × HFN nodes matrix used in the FNN approach. For each calculation, 58 nodes at a certain oscillation frequency and 81 time windows or samples (indicated by a rectangle) were used to train the FNN on 61 randomly chosen samples and to test it on the remaining 20 samples. **(C)** Three-layer FNN containing input (58 neurons), hidden (8 neurons), and output (6 neurons) layers.

To investigate the stimulus-related NTD, we used the FNN classifier trained by a standard back-propagation algorithm. The aim of this procedure was to prove whether there is a specific NTD during the 10-s interval related to the stimuli. For this purpose, we divided the observed 10-s interval into 6 ISIs, with each time sample within each ISI being provided with a class number indicating specific ISI (see Figure 4A for details). To match the stimulation structure with the dynamics of GTA measures (Figure 4B), we used the same procedure as used in the case of coupling with a 2-s moving time window and 100-ms time delay to label samples within ISIs, whereby the label was set on the time window onset. In general, there were six different classes corresponding to the six ISIs within the segment. Thereafter, we trained the FNN classifier on NTD metrics to automatically recognize the ISI classes and proved the classification accuracy (*CA*) of the network classifier. We used a three-layer FNN with an input layer, a hidden layer, and an output layer. The input layer contained 58 input neurons (corresponding to 58 electrodes at a certain frequency), the hidden layer contained 8 hidden neurons (this number of neurons was evaluated experimentally), and the output layer resulted in mostly 6 output neurons, equivalently to the number of classes (see Figure 4C). We used a hyperbolic tangent (*tanh*) transfer function: *y* = *tanh*(*x*). Like the standard logistic sigmoid function, the *tanh* function is also sigmoidal (i.e., s-shaped), but outputs values range between −1 and +1, i.e., negative and positive inputs to *tanh* are mapped to negative and positive outputs, respectively. These properties of the *tanh* transfer function make the network more stable and less likely to get “stuck” during training.

The experimental sets with 58 input variables and 81 samples were divided into the training set (75%) and the testing set (25%) consisting of 61 and 20 randomly chosen samples, respectively. The performance of the FFN classifier was determined by the *CA* as a ratio (in percent) of correctly classified items to the total number of items or classes within the testing set. The training and evaluation procedures were repeated 100 times, and we report the average *CA* of these repetitions. We tested the six GTA measures described above (*S*_*in*_, *S*_*out*_, *CC, CPL, E*_*loc*_, and *E*_*glob*_) separately for each oscillating frequency under the two oddball-task conditions (UOT and AOT). For calculations, we used the Machine Learning Toolkit from Labview (National Instruments, Munich, Germany).

### Data reduction and statistical analyses

For statistical analyses, the network vertices of 58 electrode locations oscillating at 10 different frequencies were collapsed into 5 brain sites at each frequency: F (frontal electrodes: Fp1, Fpz, Fp2, F7, …F6, F8), C (central electrodes: FC3, FC1, …, C1, Cz, C2, …, CP2, CP4), P (parieto-occipital electrodes: P7, P5, …, PO8, O1, Oz, O2), LT (left temporal electrodes: FC5, T7, C5, TP7, CP5), and RT (right temporal electrodes: FC6, T8, C6, TP8, CP6). We first analyzed the WFC and CFC connectivity strengths (ICI values) using a three-way repeated measures ANOVA with three within-subject factors Condition (REC, REO, AOT, and UOT), Site (F, C, P, LT, and RT), and Frequency (10 frequency bins). This analysis was performed separately for the WFC and CFC connectivity data determined during the entire 10-s time interval and averaged across all available segments. Next, we determined GTA measures described above for the common network using the moving window within one arbitrary chosen trial for each experimental condition. Using the nodes × time windows matrix (580 × 81, Figure 3B) described above, we calculated means and standard deviations across time and nodes. Both the means and the standard deviations (SD) for each GTA metric averaged across all time points and all nodes were then subjected to a one-way repeated measures ANOVA with the within-subject factor Condition. The NTD was assessed by calculating the numbers of dynamic states and of nodal communities, the minimal and the maximal durations of dynamic states as well as the minimal and the maximal sizes of nodal communities, which were then analyzed using a two-way repeated measures ANOVA with the two within-subject factors Condition and GTA measures (GTAs: *S*_*in*_, *S*_*out*_, *CC, CPL, E*_*loc*_, and *E*_*glob*_). A three-way repeated measures ANOVA with the three within-subject factors Condition, GTA measures, and Frequency was used to analyze the *CA* in the stimulus-related NTD approach. Greenhouse-Geisser epsilons were used in all ANOVAs for non-sphericity correction when necessary. Fischer's LSD test was employed for *post-hoc* testing.

## Results

### Synchronization patterns within and between frequencies

**Figure 5:**
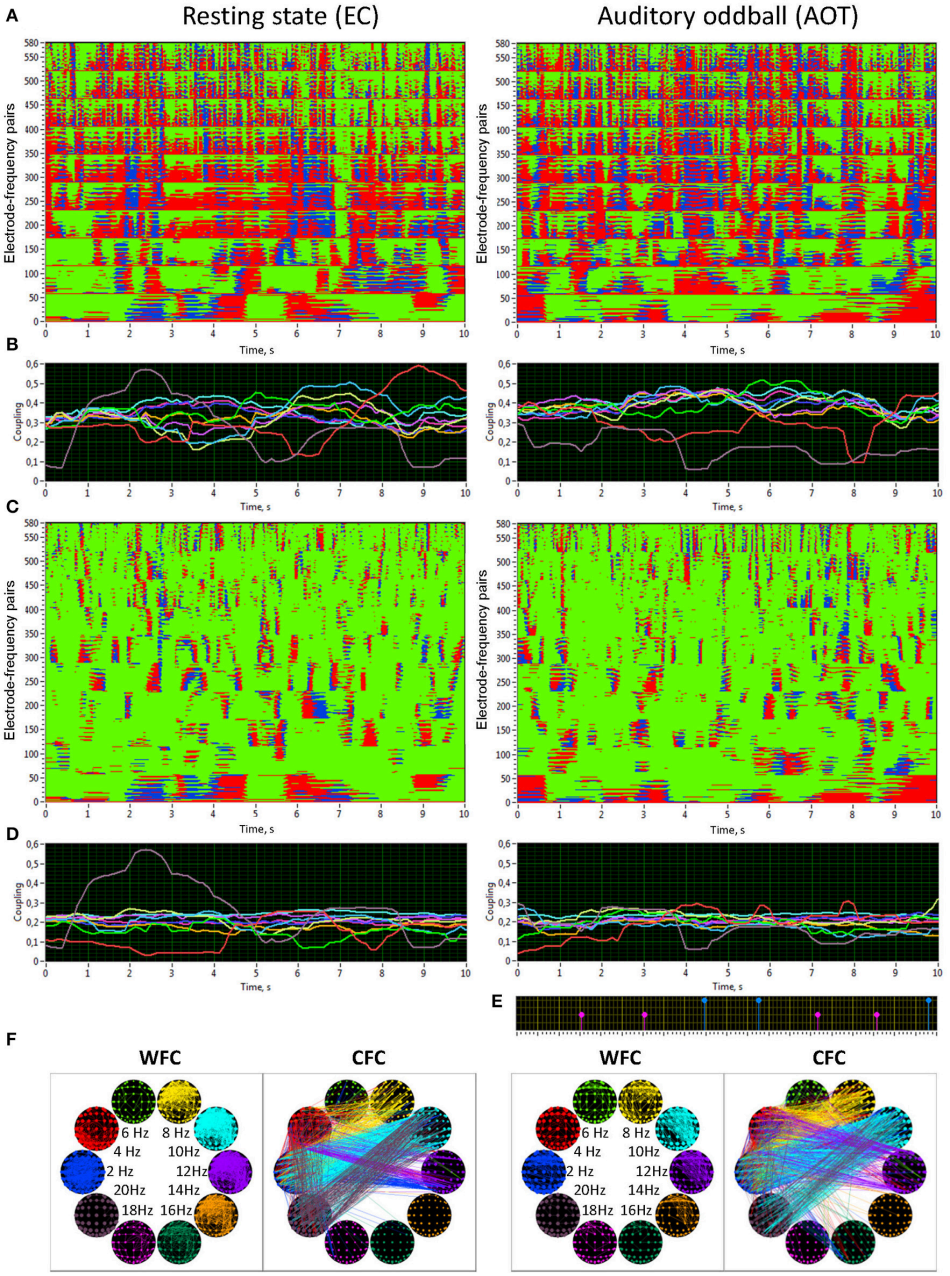
**Synchronization patterns and HFN brain maps for WFC and CFC at REC and AOT conditions both with eyes closed. (A)** Synchronization patterns for WFC from one channel (Fpz) to all other channels at 10 FOIs (58 × 10 = 580 lines) across time (10-s segment). **(B)** Time course of ICI averaged across 58 electrode pairs (Fpz to all other electrodes as presented in **A**) for the 10 FOIs (indicated by color). **(C)** Synchronization patterns for CFC from one channel (Fpz) oscillating at the frequency of 2 Hz to all other channels oscillating at 10 FOIs (58 × 10 = 580 lines) across time (10-s segment). Note that the first 58 lines are the same as in **A**, representing the coupling of 2–2 Hz, i.e., a WFC or 1:1 CFC, displayed here for comparison. **(D)** Time course of ICI averaged across 58 electrode pairs (Fpz oscillating at 2 Hz to all other electrodes as presented in **C**) for the 10 FOIs (indicated by color). **(E)** Stimulus marker in AOT condition indicating stimulus-onset. Red markers indicate deviant stimuli and blue markers indicate standard stimuli. **(F)** Brain maps of WFC (ICI > 0.55) and CFC (ICI > 0.32) at the 10 FOIs. Only the strongest connections are displayed. Note the directionality of the coupling, which is indicated by color in CFC maps; in the WFC maps the directionality of the coupling is not displayed

Figure 5 shows the synchronization patterns for WFC (Figure 5A) and CFC (Figure 5C), corresponding dynamic coupling waveforms for the 10 oscillation frequencies (Figures 5B,D), a marker indicating stimulus-onset (Figure 5E), and brain maps within and between frequencies (Figure 5F) displayed for the rest (REC) and task (AOT) conditions, both with eyes closed. Synchronization patterns for WFC at the Y-axis comprise 580 coupling traces of the Fpz electrode to all other electrodes at the 10 FOIs. Synchronization patterns for CFC are displayed for 580 coupling traces of the Fpz electrode at a 2-Hz oscillation to all other electrodes at the 10 FOIs (note that the first 58 traces in both diagrams are similar, representing coupling of 2–2 Hz). The coupling waveforms represent averages across the 58 electrodes at each frequency (Figure 5B) or combination of frequencies (Figure 5D). The brain maps of coupling calculated across the entire 10-s window are shown for WFC and CFC at the 10 different frequencies. As expected, synchronization within the frequencies is stronger than between the frequencies, but there are no recognizable differences between the conditions for both WFC and CFC.

**Figure 6:**
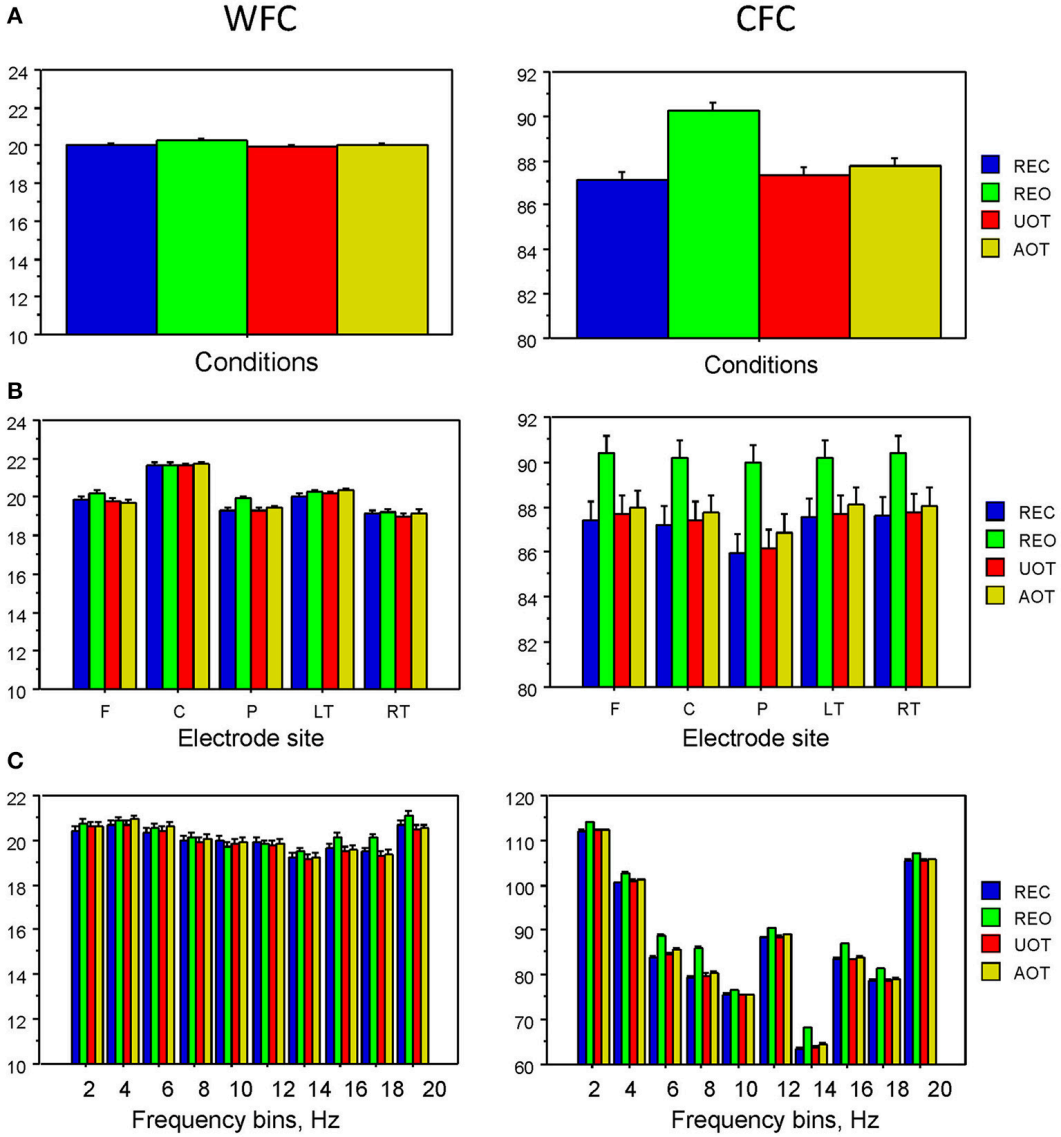
**ANOVA results for WFC and CFC. (A)** Diagrams of WFC (left) and CFC (right) under the different task conditions. **(B)** Diagrams of WFC (left) and CFC (right) for the different electrode sites under the four task conditions. **(C)** Diagrams of WFC (left) and CFC (right) for the different FOIs under the four task conditions.

Statistical analyses of WFC and CFC data using a three-way repeated measures ANOVA with the three within-subject factors Condition (REC, REO, AOT, and UOT), Site (F, C, P, LT, and RT), and Frequency (10 frequency bins) revealed a significant main effect of Condition for CFC but not for WFC (see Table 1 and Figure 6 for details). The *post-hoc* Fischer's LSD test for CFC values showed significantly higher coupling in REO than in other conditions (REO > REC, *P* < 0.0001; REO > UOT, *P* < 0.0001; REO > AOT, *P* < 0.0001), and significant differences between AOT and REC conditions (AOT > REC, *P* = 0.029). Condition differences were modulated by frequency and electrode site, as shown by the significant interactions Condition × Site, Condition × Frequency, and Condition × Site × Frequency for both CFC and WFC (see Table 1 and Figure 6). These differences were strongest at frontal and parietal sites for both WFC and CFC, and stronger in the beta frequency band for WFC and in the high theta/low alpha (e.g., 6 and 8 Hz) band for CFC.

**Table 1:** **ANOVA results for the WFC and CFC**.

**Factors**	***F*-value**	***P*-value**	**Partial eta squared**
**WITHIN-FREQUENCY COUPLING (WFC)**			
Condition	*F*_(3, 30)_ = 0.92	P = 0.44	η^2^ = 0.03
Site	*F*_(5, 145%)_ = 59.95	*P* < 0.0001	η^2^ = 0.67
Frequency	*F*_(9, 261%)_ = 24.19	*P* < 0.0001	η^2^ = 0.45
Condition × Site	*F*_(5, 145%)_ = 2.51	*P* < 0.05	η^2^ = 0.08
Condition × Frequency	*F*_(9, 261%)_ = 2.92	*P* < 0.05	η^2^ = 0.09
Site × Frequency	*F*_(5, 1305%)_ = 5.98	*P* < 0.0001	η^2^ = 0.17
Condition × Site × Frequency	*F*_(45, 1305%)_ = 1.74	P = 0.052	η^2^ = 0.06
**CROSS-FREQUENCY COUPLING (CFC)**			
Condition	*F*_(1, 29%)_ = 57.65	*P* < 0.0001	η^2^ = 0.66
Site	*F*_(5, 145%)_ = 35.31	*P* < 0.0001	η^2^ = 0.54
Frequency	*F*_(9, 261%)_ = 2094.43	*P* < 0.0001	η^2^ = 0.99
Condition × Site	*F*_(5, 145%)_ = 13.66	*P* < 0.0001	η^2^ = 0.31
Condition × Frequency	*F*_(9, 261%)_ = 20.18	*P* < 0.0001	η^2^ = 0.40
Site × Frequency	*F*_(5, 1305%)_ = 15.00	*P* < 0.0001	η^2^ = 0.33
Condition × Site × Frequency	*F*_(45, 1305%)_ = 2.62	*P* < 0.0001	η^2^ = 0.08

### Network structure and network dynamics

For representation of network structures and network dynamics, network coupling and corresponding network structures were determined for each moving window of 2 s with a time delay of 100 ms during the 10-s time period (81 windows in total). To determine the network properties, we set the connectivity threshold to 0.26, which was always higher than the significance level determined by the surrogate data procedure, that is, networks at this threshold level always included significant connections. At this threshold, the cost level of the networks (i.e., the ratio of the number of actual connections divided by the maximum possible number of connections in the network) was approximately 20%, corresponding to high sparsity networks and allowing more accurate examination of the network topology.

**Figure 7:**
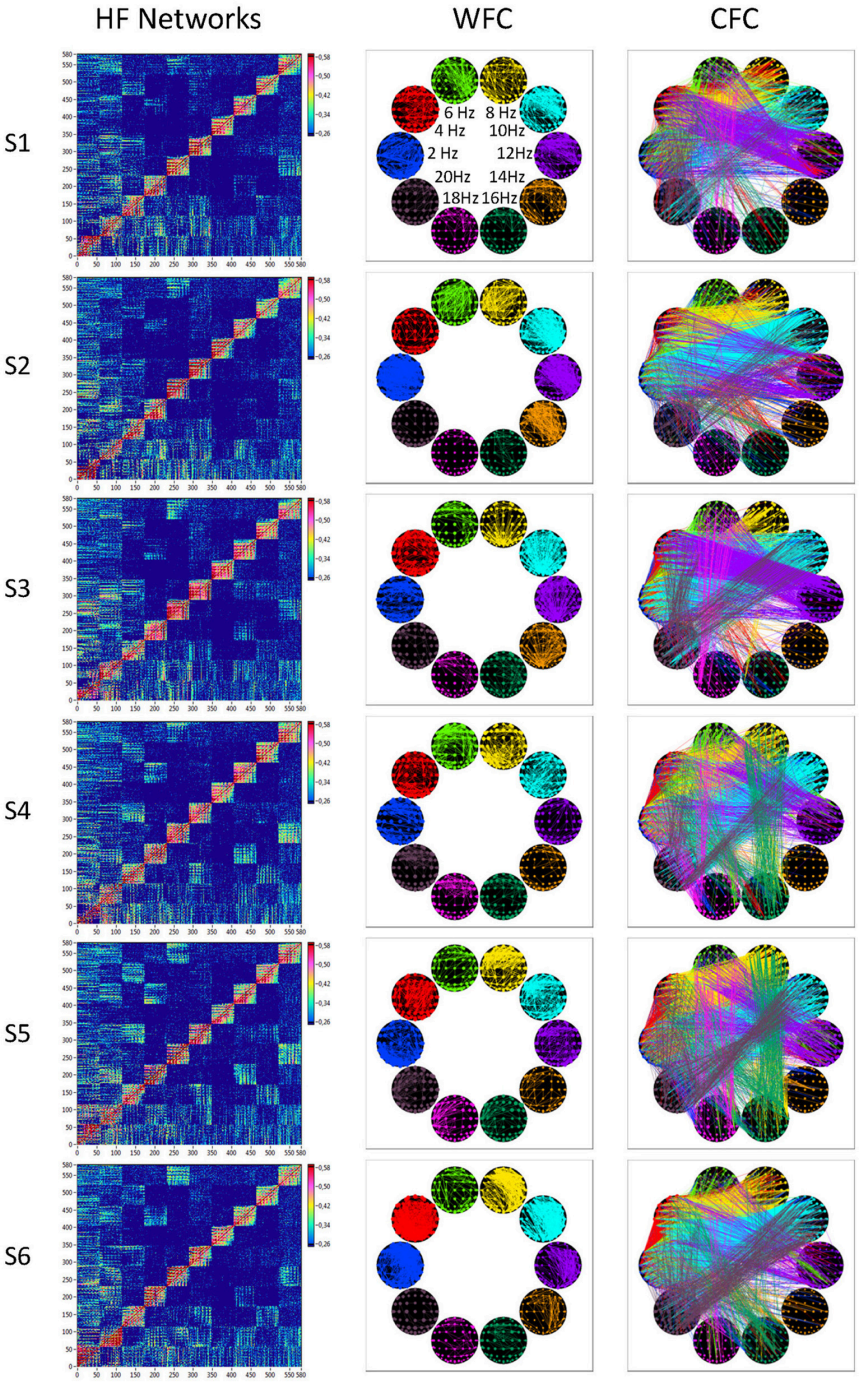
**HFNs and corresponding brain maps of WFC and CFC**. The different HFNs and the brain maps of WFC (ICI > 0.65) and CFC (ICI > 0.40) are displayed for the six time windows related to the six stimulus onsets in the AOT condition.

**Figure 8:**
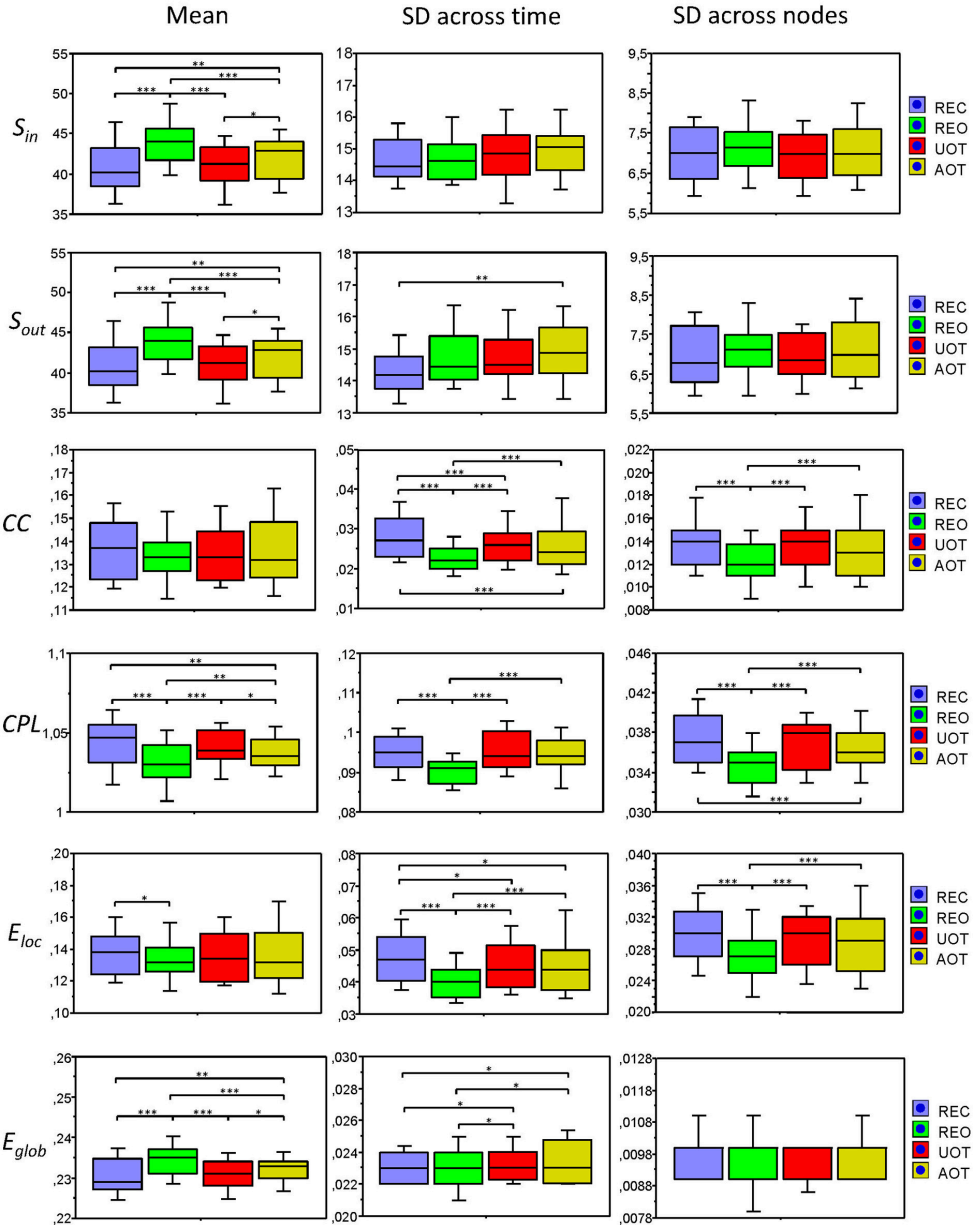
**Box plots of the means and standard deviations of the six GTA measures across the four task conditions**. Box plots of the means calculated across all network nodes and then averaged across the 81 time windows. SD was calculated in two different ways: (1) across all network nodes at each time window and then averaged across the 81 time windows, and (2) across all time windows at each node and then averaged across the 580 network nodes. The first represents the variability of nodes' magnitude within the network in time, and the second reflects the variability or dynamic changes of nodes. The data are presented for the six GTA measures and the four task conditions: *S*_*in*_, In-Strength; *S*_*out*_, Out-Strength; *CC*, Clustering Coefficient; *CPL*, Characteristic Path Length; *E*_*loc*_, Local Efficiency; and *E*_*glob*_, Global Efficiency. Significant differences between conditions are indicated as follows: ^*^p < 0.05, ^**^p < 0.01, ^***^p < 0.001.

In Figure 7, we present the common network structures based on WFC and CFC depicted on corresponding brain maps for six different time windows around the six stimulus events in the AOT condition. It can be seen that both WFC and CFC, and corresponding network structures change across time with different connectivity patterns within and between the different frequencies. For the investigation of the network topology and its changes in time, we determined in- and out-strengths, clustering coefficient and path length as well as local and global efficiency for each node in the network and each time window. We initially calculated the means (M) and standard deviations (SD) both across time windows and across nodes. For statistical analyses, we averaged and subjected them to a one-way repeated measures ANOVA with a within-subject factor condition. If the average mean provided the same results for both averaging procedures (across time and nodes), the average standard deviation was different. Results of these analyses are summarized in Table 2 and Figure 8. It can be seen that both the mean and SD differentiate well between conditions. REO and AOT conditions were characterized by greater strength and shorter path length as well as stronger *E*_*glob*_; *E*_*loc*_ was higher in the REC compared to the REO condition. SD calculated both across nodes and time windows was mostly lowest in the REO condition (see Figure 8 for details).

**Table 2:** **ANOVA results for the mean (M) and standard deviation (SD) of the six GTA measures**.

**GTA measures**	***F*-value**	***P*-value**	**Partial eta squared**
**MEAN (M)**			
*S_in_*	*F*_(3, 90%)_ = 27.16	*P* < 0.0001	η^2^ = 0.48
*S_out_*	*F*_(3, 90%)_ = 27.16	*P* < 0.0001	η^2^ = 0.48
*CC*	*F*_(3, 90%)_ = 1.09	*P* = 0.35	η^2^ = 0.04
*CPL*	*F*_(3, 90%)_ = 12.67	*P* < 0.0001	η^2^ = 0.30
*E_loc_*	*F*_(3, 90%)_ = 1.69	*P* = 0.18	η^2^ = 0.05
*E_glob_*	*F*_(3, 90%)_ = 17.35	*P* < 0.0001	η^2^ = 0.37
**STANDARD DEVIATION (SD) ACROSS TIME**			
*S_in_*	*F*_(3, 90%)_ = 1.04	*P* = 0.38	η^2^ = 0.03
*S_out_*	*F*_(3, 90%)_ = 2.81	*P* < 0.05	η^2^ = 0.09
*CC*	*F*_(3, 90%)_ = 13.38	*P* < 0.0001	η^2^ = 0.31
*CPL*	*F*_(3, 90%)_ = 11.03	*P* < 0.0001	η^2^ = 0.27
*E_loc_*	*F*_(3, 90%)_ = 14.07	*P* < 0.0001	η^2^ = 0.32
*E_glob_*	*F*_(3, 90%)_ = 3.44	*P* < 0.05	η^2^ = 0.10
**STANDARD DEVIATION (SD) ACROSS NODES**			
*S_in_*	*F*_(3, 90%)_ = 1.86	*P* = 0.14	η^2^ = 0.06
*S_out_*	*F*_(3, 90%)_ = 1.89	*P* = 0.14	η^2^ = 0.06
*CC*	*F*_(3, 90%)_ = 5.35	*P* < 0.005	η^2^ = 0.15
*CPL*	*F*_(3, 90%)_ = 11.25	*P* < 0.0001	η^2^ = 0.27
*E_loc_*	*F*_(3, 90%)_ = 7.59	*P* < 0.0001	η^2^ = 0.20
*E_glob_*	*F*_(3, 90%)_ = 1.53	*P* = 0.21	η^2^ = 0.05

S_in_, In-Strength; S_out_, Out-Strength; CC, Clustering Coefficient; CPL, Characteristic Path Length; E_loc_, Local Efficiency; E_glob_, Global Efficiency.

**Figure 9:**
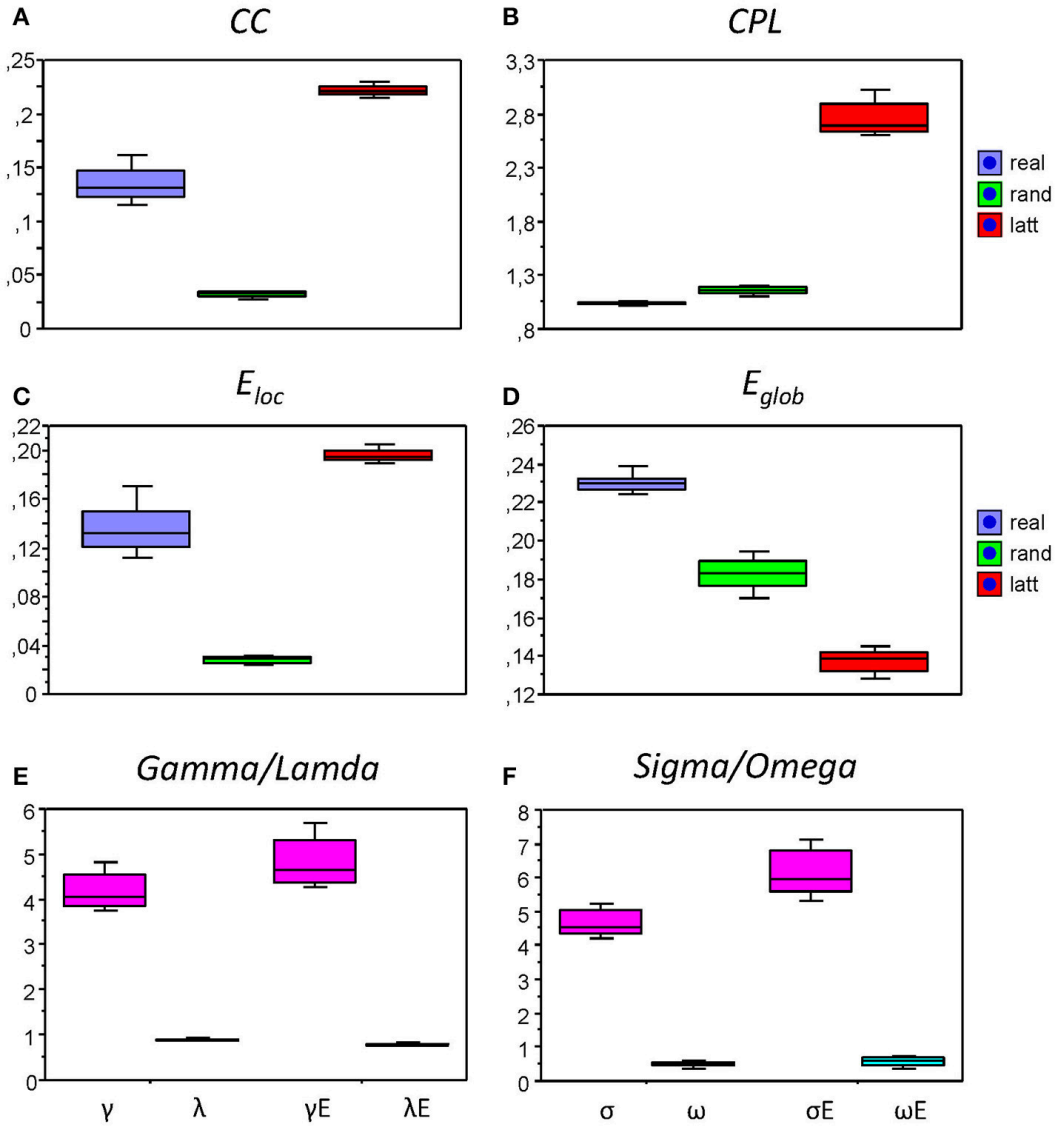
**Box plots of the mean of the small-world metric for the real and control networks (random and regular/lattice). (A)** Clustering Coefficient (*CC*); **(B)** Characteristic Path Length (*CPL*); **(C)** Local efficiency (*E*_*loc*_); **(D)** Global efficiency (*E*_*glob*_); **(E)** Normalized Clustering Coefficient (γ = *CC*_*real*_/*CC*_*rand*_) and Characteristic Path Length (λ = *CPL*_*real*_/*CPL*_*rand*_) as well as normalized Local Efficiency (γ*E* = *E*_*loc*(*real*)_/*E*_*loc*(*rand*)_) and Global Efficiency (λ*E* = *E*_*glob*(*real*)_/*E*_*glob*(*rand*)_); **(F)** Small-world coefficients: σ, ω, σ*E*, and ω*E*.

We also compared the topology of our real networks with that of the regular and random networks having the same number of nodes and edges (see Method Section for details), and tested whether our real HFNs were SWNs and how they were positioned in topological space compared to regular and random networks. To do this, we calculated average GTA measures (*CC, CPL, E*_*loc*_, and *E*_*glob*_) across all nodes and time windows for the three network types (real, lattice, and random) and then determined corresponding metrics related to the random networks (γ, λ, γ*E*, and λ*E*) and corresponding small-world coefficients (σ, ω, σ*E*, and ω*E*). The graph metrics for the control networks (regular and random) and the respective topology changes were determined only for the AOT condition, because we expected other conditions to show similar relationships between real and control networks. All these network topology metrics are presented in Figure 8 as box plots. As expected, *CC* (Figure 9A), and respectively *E*_*loc*_ (Figure 9C), of the real networks are higher than those of random networks and lower than those of regular or lattice networks. *CPL* (Figure 9B) is shorter and *E*_*glob*_ (Figure 9D) correspondingly higher in real networks as compared to both control networks, especially, as compared to the lattice network with very long CPL and very low *E*_*glob*_. All the differences between the different network types were highly significant (*p* < 0.0001). High local clustering (γ and γ*E*) and short global path length (λ and λ*E*) normalized to the random network in this case (Figure 9E) indicate that the networks are SWNs. This is also confirmed by the small-world coefficients (Figure 9F) with sigma (σ and σ*E*) much higher than 1 and omega (ω and ω*E*) ranging between 0 and 1. Positive values of omega small-world coefficients indicate that real networks are SWNs with more random characteristics.

### Temporal and nodal network similarity

**Figure 10:**
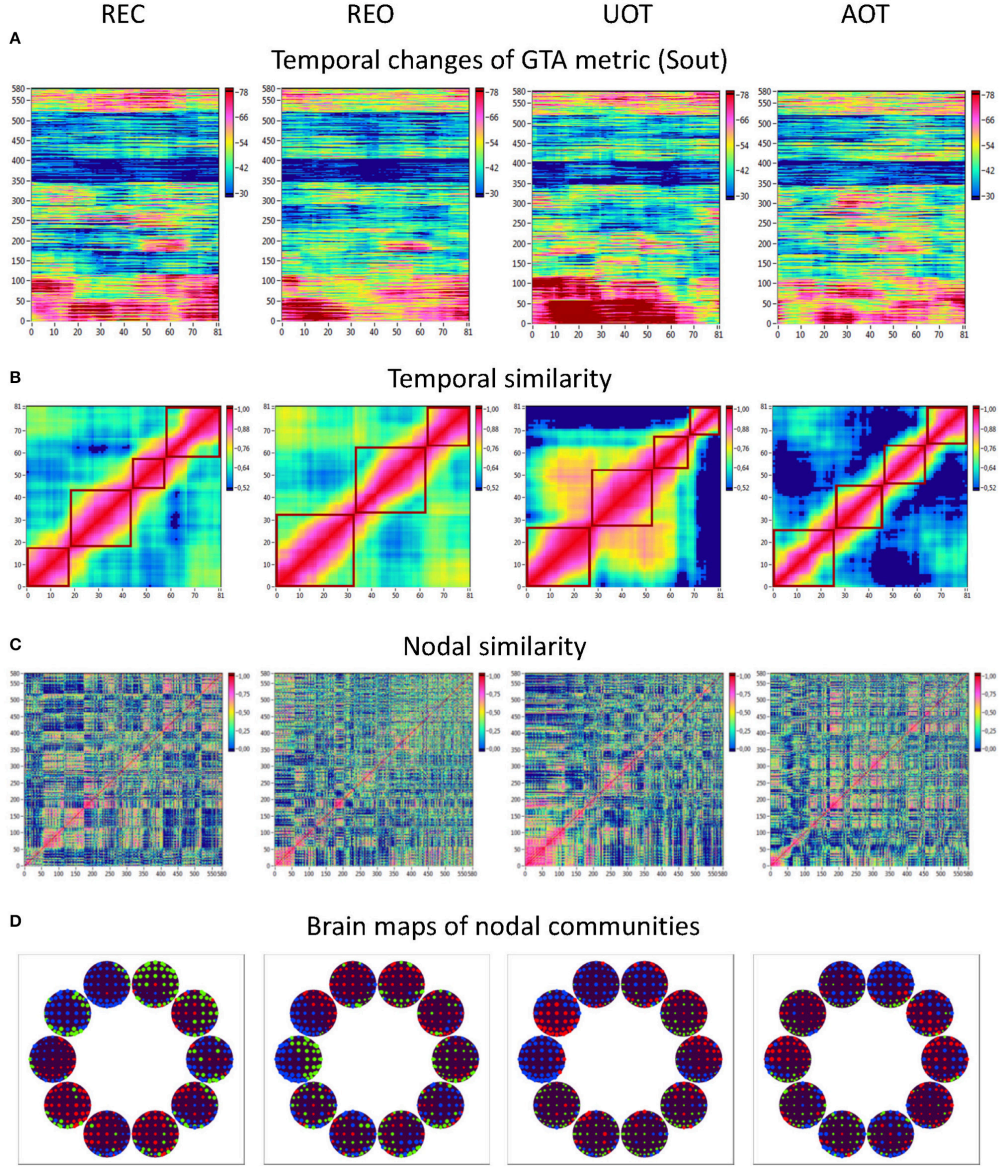
**HFN dynamics with temporal and nodal similarity during resting state (REC and REO) and oddball task (UOT and AOT). (A)** Temporal changes of a GTA metric (*S*_*out*_) calculated for each HFN node and resulting in a time window × GTA metric matrix (81 × 580); **(B)** The temporal similarity matrix was built by calculation of Pearson's product correlation among the consecutive vertical lines in the previous matrix (in **A**). Using modularity analysis, dynamic states were determined in the temporal similarity matrix indicating a similar time course of a GTA metric of HFN. Different dynamic states are presented as quadrants along the diagonal of the temporal similarity matrix. **(C)** Nodal similarity matrix was built by calculation of Pearson's product correlation among the consecutive horizontal lines in the previous 81 × 580 matrix (in **A**). **(D)** Brain maps of nodal communities determined using Louvain modularity analysis. Nodal communities are indicated by color.

**Figure 11:**
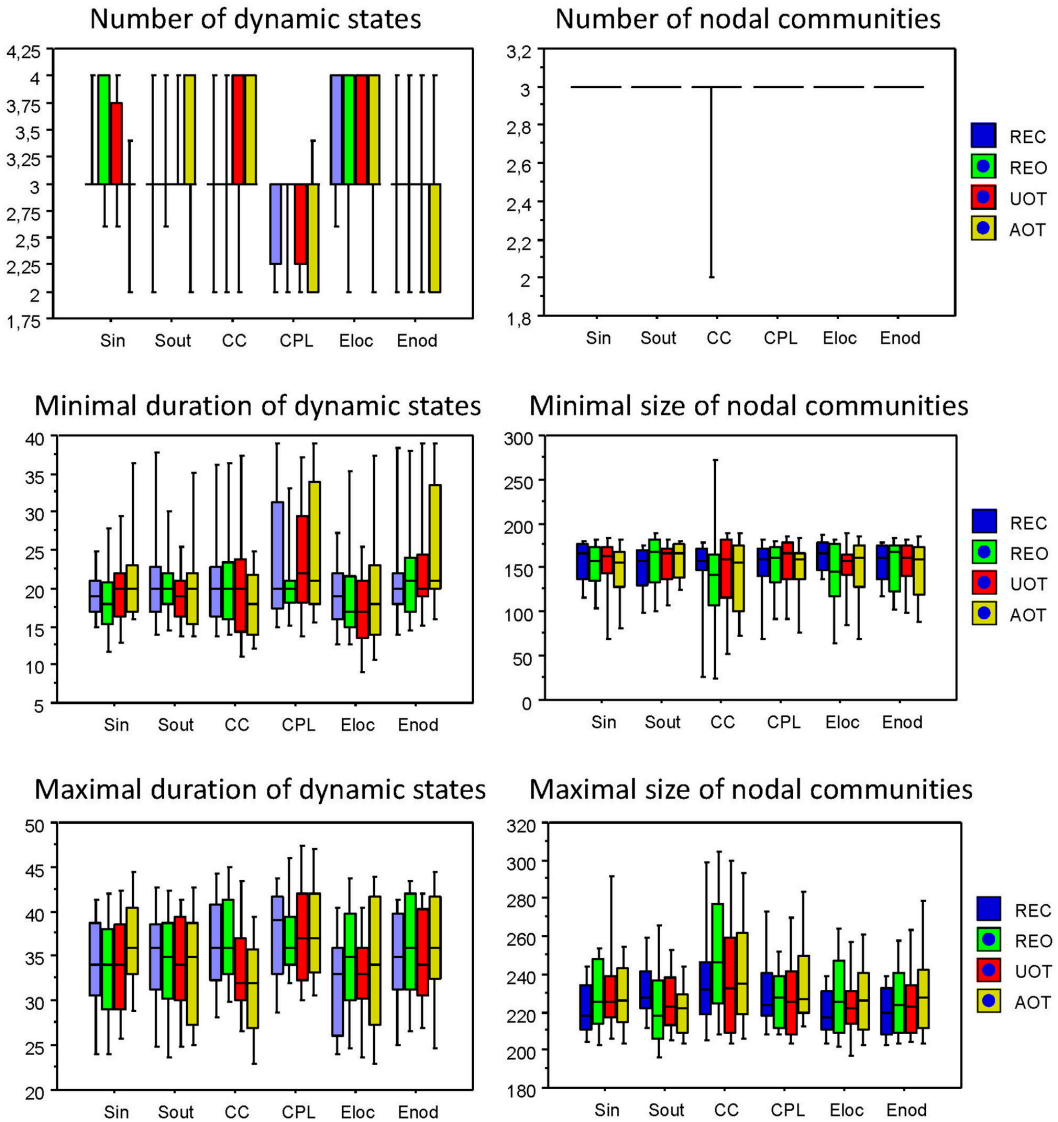
**Box plots of the characteristics of dynamic states and nodal communities**. Characteristics of dynamic states are presented on the left: number of dynamic states, minimal duration of dynamic states, and maximal duration of dynamic states. Characteristics of nodal communities presented on the right: number of nodal communities, minimal size of nodal communities, and maximal size of nodal communities.

Using the HFN topology changes described above, we built a nodes × time windows matrix (580 × 81) for each network metric and analyzed it for temporal network similarity (correlations among consecutive time windows) and for spatial or nodal network similarity (correlations among consecutive nodes). In both cases, similarity was determined by Pearson's product correlation. Resulting correlation matrices were used for (1) identification of sequences of coherent states (81 × 81 correlation matrix indicating temporal network similarity), and (2) identification of node communities remaining stable or similar across time (580 × 580 correlation matrix indicating nodal network similarity). We used modularity analyses in both cases. Given that nodal network similarity entails both positive and negative values, we used the Louvain modularity algorithm in this case. Dynamic changes of out-strengths for the 580 nodes across the 81 time windows and corresponding correlation matrices for the four experimental conditions are presented in Figure 10. Similarity matrices show that network out-strengths (and also other topological network measures) vary both across time and space/nodes (Figure 10A). Temporal similarity was generally very high but modularity analyses were able to distinguish different regions or dynamical states as indicated by the quadrants across the diagonal in temporal similarity matrices (Figure 10B). Nodal similarity varied much more strongly than temporal similarity (Figure 10C), although there are mostly three blocks of nodes that varied differently in time, as indicated by color in the brain maps of nodal communities (Figure 10D). Interestingly, different nodal communities had their agents mostly (with some exceptions) at all oscillation frequencies, but at the same time there were specific brain regions oscillating at certain frequencies that were organized into specific communities. In the REC condition, for example, the blue community mostly comprises nodes in the delta-theta (2–6 Hz) and also high alpha (12 Hz) frequency ranges, whereas the green community mostly comprises nodes in the delta (4 Hz) and low alpha (8 and 10 Hz) frequency, and the red community mainly comprises nodes in the beta frequency (14–20 Hz) with a relatively strong participation of nodes oscillating at delta (2 Hz) and alpha (10 Hz) frequencies. This community structure differed as a function of condition (cf. Figure 10D for details). In addition, we calculated the number and the duration/size of the dynamic states and nodal communities for each condition. The data for all GTA measures and conditions are summarized in Tables 3, 4 for temporal and nodal similarity, respectively. Figure 11 presents box plots of these data. It can be seen that the number of dynamic states varied between two and four, with a mode at three, whereby some measures (*S*_*in*_, *S*_*out*_, *CC*, and especially *E*_*loc*_) tend to indicate four states, whereas other measures (e.g., *CPL*) point to two states. The minimal duration of dynamic states varied at around 20 time windows, which corresponds to ~2.5 s. The maximal duration of dynamic states varied around 35 time widows, which corresponds to approximately 4.3 s. Statistical analyses showed reliable differences among the GTA measures but did not reveal any reliable differences between conditions (see Table 5 for details). The numbers of nodal communities practically did not vary and is mostly equal to three. The minimal nodal community size varied around 150 nodes, and the maximal nodal community size varied around 220–230 nodes. *CC* showed the largest nodal community, comprising on average 243 nodes. Again, statistical analyses showed reliable differences among the GTA measures, which were restricted to the maximal nodal community size, but no reliable condition differences (see Table 5 for details).

**Table 3:** **Mean (M) and standard deviation (SD) for the number of dynamic states and their minimal and maximal duration across the GTA measures and task conditions**.

**Measure**	**Condition**	**Number of states**		**Minimal duration**		**Maximal duration**	
		**M**	**SD**	**M**	**SD**	**M**	**SD**
*S_in_*	REC	3.1	0.5	20.0	4.9	34.1	6.4
	REO	3.2	0.6	18.8	6.9	33.8	6.7
	UOT	3.2	0.6	20.1	6.9	34.0	6.2
	AOT	2.9	0.5	22.5	7.2	36.3	6.0
*S_out_*	REC	3.0	0.6	21.7	7.9	34.8	6.4
	REO	3.1	0.6	20.9	6.2	34.1	6.4
	UOT	3.1	0.5	19.8	6.1	33.7	6.1
	AOT	3.2	0.6	20.5	7.7	33.8	6.6
*CC*	REC	2.9	0.6	21.8	8.1	36.3	6.0
	REO	2.9	0.6	21.6	8.7	36.5	6.1
	UOT	3.1	0.7	21.4	9.3	33.8	6.0
	AOT	3.3	0.6	18.7	6.3	31.6	6.3
*CPL*	REC	2.8	0.5	23.5	9.3	37.2	6.0
	REO	2.9	0.5	21.1	6.4	37.0	5.9
	UOT	2.8	0.5	23.5	8.1	37.5	6.3
	AOT	2.7	0.6	24.9	9.4	37.5	6.6
*E_loc_*	REC	3.3	0.6	20.0	6.9	32.3	6.4
	REO	3.2	0.8	19.5	8.2	34.5	7.3
	UOT	3.3	0.6	18.2	7.2	33.0	5.9
	AOT	3.1	0.7	20.5	9.4	34.4	7.6
*E_glob_*	REC	3.0	0.5	21.5	7.7	34.6	5.7
	REO	2.9	0.6	22.7	8.3	36.3	7.0
	UOT	3.0	0.6	22.4	8.2	34.2	6.0
	AOT	2.8	0.6	24.6	8.5	36.3	6.7

The minimal and maximal duration was calculated as the number of time windows.

**Table 4:** **Mean (M) and standard deviation (SD) for the numbers of nodal communities and their minimal and maximal nodal sizes across the GTA measures and task conditions**.

**Measure**	**Condition**	**Number of blocks**		**Minimal size**		**Maximal size**	
		**M**	**SD**	**M**	**SD**	**M**	**SD**
*S_in_*	REC	3.0	0.2	156.3	25.9	222.0	17.2
	REO	3.0	0.0	149.4	36.0	229.1	21.1
	UOT	3.0	0.2	150.8	50.0	233.7	28.3
	aot	3.0	0.0	143.8	39.1	230.2	22.8
*S_out_*	REC	3.0	0.0	146.8	29.4	232.5	17.9
	REO	3.0	0.2	152.1	42.5	222.5	28.3
	UOT	3.0	0.0	154.7	28.8	226.4	18.5
	AOT	3.0	0.0	155.5	29.5	221.7	18.0
*CC*	REC	3.0	0.0	140.5	53.5	238.2	30.9
	REO	2.9	0.3	141.4	71.4	250.6	35.5
	UOT	2.9	0.3	145.5	62.1	240.0	34.4
	AOT	2.9	0.3	142.0	55.4	242.5	32.1
*CPL*	REC	3.0	0.0	145.0	43.8	231.7	24.3
	REO	3.0	0.0	145.2	42.7	229.5	22.6
	UOT	3.0	0.3	152.8	48.3	229.0	26.9
	AOT	2.9	0.3	148.8	52.1	237.7	28.9
*E_loc_*	REC	3.0	0.2	162.7	33.6	223.8	24.5
	REO	3.0	0.2	136.3	46.8	230.0	30.1
	UOT	3.0	0.0	149.1	33.9	224.5	20.4
	AOT	3.0	0.0	143.4	49.0	231.3	29.0
*E_glob_*	REC	3.0	0.2	155.2	23.0	221.1	14.6
	REO	3.0	0.0	147.8	39.1	227.1	21.3
	UOT	2.9	0.4	163.4	85.8	236.4	67.1
	AOT	2.9	0.5	142.2	48.5	250.7	90.3

The minimal and maximal nodal size was calculated as the number of nodes in the corresponding nodal community.

**Table 5:** **ANOVA results for the different measures of dynamic states and nodal communities**.

**Factors**	***F*-value**	***P*-value**	**Partial eta squared**
**NUMBER OF DYNAMIC STATES**			
Condition	*F*_(3, 90%)_ = 0.20	*P* = 0.88	η^2^ = 0.01
GTAs	*F*_(5, 150%)_ = 7.65	*P* < 0.0001	η^2^ = 0.20
Condition × GTAs	*F*_(15, 450%)_ = 1.54	*P* = 0.13	η^2^ = 0.05
**MINIMAL DURATION OF THE DYNAMIC STATES**			
Condition	*F*_(3, 90%)_ = 0.58	*P* = 0.62	η^2^ = 0.02
GTAs	*F*_(5, 150%)_ = 3.89	*P* < 0.01	η^2^ = 0.12
Condition × GTAs	*F*_(15, 450%)_ = 1.11	*P* = 0.36	η^2^ = 0.04
**MAXIMAL DURATION OF THE DYNAMIC STATES**			
Condition	*F*_(3, 90%)_ = 0.40	*P* = 0.73	η^2^ = 0.01
GTAs	*F*_(5, 150%)_ = 6.22	*P* < 0.0001	η^2^ = 0.17
Condition × GTAs	*F*_(15, 450%)_ = 1.49	*P* = 0.15	η^2^ = 0.05
**NUMBER OF NODAL COMMUNITIES**			
Condition	*F*_(3, 90%)_ = 2.35	*P* = 0.090	η^2^ = 0.07
GTAs	*F*_(5, 150%)_ = 2.22	*P* = 0.087	η^2^ = 0.07
Condition × GTAs	*F*_(15, 450%)_ = 1.28	*P* = 0.28	η^2^ = 0.04
**MINIMAL SIZE OF THE NODAL COMMUNITIES**			
Condition	*F*_(3, 90%)_ = 0.69	*P* = 0.54	η^2^ = 0.02
GTAs	*F*_(5, 150%)_ = 0.66	*P* = 0.61	η^2^ = 0.02
Condition × GTAs	*F*_(15, 450%)_ = 0.69	*P* = 0.71	η^2^ = 0.02
**MAXIMAL SIZE OF THE NODAL COMMUNITIES**			
Condition	*F*_(3, 90%)_ = 1.08	*P* = 0.35	η^2^ = 0.04
GTAs	*F*_(5, 150%)_ = 4.00	*P* < 0.05	η^2^ = 0.12
Condition × GTAs	*F*_(15, 450%)_ = 1.35	*P* = 0.25	η^2^ = 0.04

GTAs = GTA measures.

### Stimuli-related network dynamics

**Figure 12:**
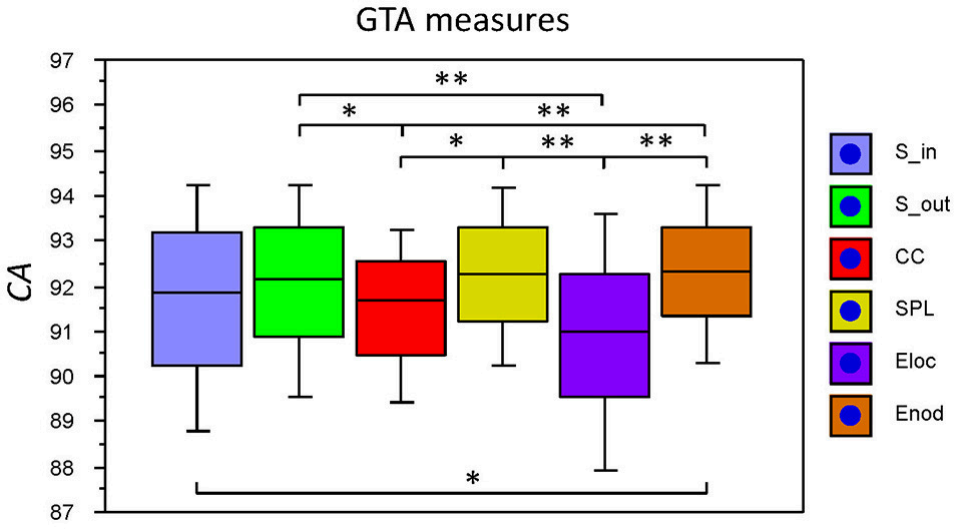
**Box plots of the *CA* for FNN performance across different GTA measures**. The data are presented for the six GTA measures: *S*_*in*_, In-Strength; *S*_*out*_, Out-Strength; *CC,* Clustering Coefficient; *CPL*, Characteristic Path Length; *E*_*loc*_, Local Efficiency; and *E*_*glob*_, Global Efficiency. Significant differences between conditions are indicated as follows: ^*^p < 0.05, ^**^p < 0.01.

The fact that conditions did not differ for both dynamic states (number and duration) and nodal communities (number and size) indicates that there are certain invariances in network dynamics. Moreover, the duration of dynamic states, which is equal during the oddball task and resting state, is longer than the ISI during the task. In other words, this dynamics is not related to the stimulus impact. This poses a question: Does another network dynamics exist that would be able to describe the network changes related to the task stimulation? To answer this question, we labeled the ISIs during the 10-s segment with different levels that serve as different classes for the FNN classifier. The experimental sets with 58 input variables and 81 samples were divided into the training and the testing set. The performance of the FFN classifier was determined by the *CA* as a ratio (in percent) of correctly classified items to the total number of items or classes within the testing set. We tested the six GTA measures described above (*S*_*in*_, *S*_*out*_, *CC, CPL, E*_*loc*_, and *E*_*glob*_) under two oddball-task conditions (UOT and AOT) separately for the 10 oscillating frequencies. We found that the FFN classifier was able to differentiate between different ISIs with a total accuracy of 91.6%. A three-way repeated measures ANOVA with the three within-subject factors Condition, GTA measures, and Frequency showed a significant main effect GTA measures only [*F*_(5, 150)_ = 3.74, *P* < 0.01], with lower *CA* for *CC* and *E*_*loc*_ compared to the other GTA measures (see Figure 12 for details). There were no reliable differences between frequencies or conditions. In all cases, there was a high classification accuracy indicating that there are dynamic states or dynamic patterns that describe neuronal network changes related to processing of stimuli, which apparently differ from the dynamics described above.

## Discussion

Neural network oscillations are a fundamental mechanism for the establishment of precise spatiotemporal relationships between neural responses that are in turn relevant for cognition, memory, perception, and consciousness. When neurons discharge, the subsequent oscillatory activity propagates through the network recruiting other brain regions, thereby dynamically binding widely distributed sets of neurons into functionally coherent ensembles, hypothesized to represent neural correlates of a cognitive or behavioral content (Singer, 1999). As the transient wave evolves, it establishes a spatiotemporal pattern characteristic for cognitive processes (Bressler, 1995; Roelfsema et al., 1997), sensory (Engel et al., 1991), motor and sensorimotor tasks (Kelso, 1995), resting state (Allen et al., 2014; Hansen et al., 2015), and stimulation paradigms (Spiegler et al., 2016). Simple activation paradigms lack the functional complexity to explain the richness of observed spatiotemporal behaviors linked to these brain dynamics (Bressler, 1995). Mechanisms based on organizing oscillatory activity into network patterns have been proposed, including synchronization and some derivatives such as communication through coherence (Fries, 2005; Bastos et al., 2014). These mechanisms have been limited so far to interference at one frequency. Here we have proposed a generalization of these mechanisms extending oscillatory interference patterns across frequencies binding information into HFN. We demonstrate the existence of HFNs, which are constructed by WFC and CFC and possess SWN topology with different NTD. The dynamics are characterized by variances and invariances during resting state and stimulus processing with and without attentional load (cf. also Sleimen-Malkoun et al., 2015). Importantly, dynamic network reorganization differentially affects different network topologies indicating that these dynamic changes are heterogeneous.

More specifically, we systematically examined network structure and network dynamics during rest with eyes closed and eyes open, and during auditory oddball performance. The main findings are that: (a) in general, CFC better differentiates between task conditions than WFC; (b) HFNs constructed in this way possess small-world topology with a slight tendency to random characteristics; (c) mean and standard deviation of GTA metrics calculated across time and different HFN nodes indicate temporal and spatial differences between metrics; (d) temporal changes of network topology reveal relatively high network similarity, however, several dynamic states could be distinguished; (e) nodal similarity indicates several (mostly 3) nodal communities showing similar NTD within these communities; (f) in addition to dynamic states, which were invariant across conditions, we found stimulus-related dynamics that referred to stimulation structure.

The fact that CFC was stronger in the REO than in other conditions was not unexpected, although there is evidence that linear and non-linear coupling decreases during resting state with eyes open as compared with eyes closed (Müller and Lindenberger, 2012; Tan et al., 2013). However, in a previous study (Jirsa and Müller, 2013), it has been found that at least half of the connections in the case of delta-alpha phase to phase coupling were stronger in the REO condition than in the REC condition. It should be noted here that WFC only showed significant interactions related to the factor condition, indicating greater strength in the REO condition, as compared to the rest, only in the beta frequency range and only fronto-parietally. Interestingly, fMRI studies (Yan et al., 2009) showed higher functional connectivity in the DMN (Default Mode Network) during resting state with eyes open as compared with eyes closed and also a stronger correlation between DMN and EEG alpha activity (Mo et al., 2013). Moreover, the strongest differences in functional connectivity among the resting state conditions (EO > EC) have been found in the PCC (posterior cingulate cortex) and the MPFC (medial prefrontal cortex) regions within the DMN (Yan et al., 2009). Notably, the strongest differences between REO and REC conditions for both WFC and CFC in our study were found in parietal and also frontal brain regions. Given the relatively low spatial resolution of EEG, we can only speculate about the neuronal circuitry contributing to these resting state differences. It has been suggested that PCC is associated with the general monitoring of sensory information (Yan et al., 2009), the flow of which is reduced during eyes closed, apparently leading to WFC and CFC reduction during REC or enhancement during REO.

Using WFC and CFC, we constructed HFN and investigated network topology and dynamic changes of this topology across time. To investigate the small-world properties of the HFNs, we compared their *CC* and *CPL* as well as *E*_*loc*_ and *E*_*glob*_ to those of regular lattices and random graphs with the same numbers of nodes and mean degrees as our real networks, and calculated two different small-worldness coefficients (i.e., σ and ω as well as σ*E* and ω*E*). In general, random networks have a low average clustering coefficient, whereas complex or SWNs have a high clustering coefficient (associated with the high local efficiency of information transfer and robustness), which is, however, much lower than that in regular networks. Random and SWNs have a short *CPL* (high global efficiency of parallel information transfer), whereas regular networks (e.g., lattices) have a long *CPL* and low global efficiency of parallel information transfer, respectively. We have shown that HFNs are fully in line with the topological characteristics of SWNs providing high local and global efficiency, supporting segregation and integration of neural processes. In addition, the small-world coefficient σ (and σ*E*), which was much greater than one, indicated that HFNs correspond to SWNs. In line with a study on FCD reporting small-worldness of time-variant networks (Chavez et al., 2010), we regard this as a remarkable result indicating permanent network optimization by a dynamic reconfiguration of network connections. The fact that the small-worldness coefficient ω (and also ω*E*) lies in the positive range indicates that HFNs are SWNs characterized by a topology with a slight tendency to random characteristics (cf. Telesford et al., 2011). This randomness of HFNs is conditioned above all by long-range connections, with low-frequency oscillations (delta and to some extent theta and alpha) playing a leading role. HFNs are organized in such a way that if there is WFC only, such a network would be akin to a regular network, and increasing CFC would increase its randomness. SWN would represent a balance between WFC and CFC. The positive small-worldness coefficient ω indicates a slight shift in the balance to CFC.

It has been shown that these networks are characterized by greater strength and the shortest path length as well as by enhanced nodal or global efficiency during REO and also during AOT. In other words, brain activation through opening the eyes (REO) or increasing attentional load (AOT) evokes stronger connectivity and also stronger integration processes in the brain. Interestingly, this activation reduces variability (SD) of topological characteristics in the REO but not in the AOT condition. Thus, stimulus processing is not only characterized by enhanced connectivity and efficiency but also by enhanced variability, at least as compared to the REC condition regarding out-strength and to the REO condition regarding other GTA measures. In our previous study (Müller and Lindenberger, 2012), we found an increase in nonlinear coupling during stimulus processing accompanied by complexity reduction, and an increase in complexity in resting state with eyes open as compared with eyes closed (compare also Mayer-Kress and Layne, 1987; Rapp et al., 1989; Stam et al., 1994; Müller et al., 2003a,b; Sleimen-Malkoun et al., 2015). How enhanced variability of coupling strength and of other network topology measures are related to complexity reduction during stimulus processing remains to be seen.

To further investigate HFN dynamics, we calculated temporal and nodal network similarity using a network nodes × time windows matrix (580 × 81) for all GTA measures. We found that temporal similarity was generally very high but modularity analyses of similarity (correlation) matrices were able to distinguish different regions or dynamical states that are separated through phase transitions. It has been shown that the number of dynamic states varies between two and four with a preferential number of three, with some measures (*S-out, CC*, and especially *E*_*loc*_) being characterized by four states, and others (e.g., *CPL*) characterized by only two states. The minimal duration of dynamic states corresponds to approximately 2.5 s, whereas the maximal duration of dynamic states corresponds to approximately 4.3 s. These dynamics were invariant across conditions but showed significant differences in minimal and maximal duration and the numbers of dynamic states between the different GTA measures. This is a remarkable result, which indicates that different HFN topologies can have different temporal patterns, which appear to depend on dynamic changes of network configuration or network reorganization (e.g., changes in *CC* do not obviously coincide in time with changes in *CPL* or other metrics). The number of nodal communities showing similar network dynamics within communities practically did not vary and is mostly equaled 3. The minimal nodal community size varies around 150 nodes, and the maximal nodal community size varies around 220–230 nodes. These variations in the number of nodes of nodal communities were also invariant across conditions. Most interestingly, nodal communities comprise nodes with different oscillation frequencies and also with different electrode sites that exhibit similar network dynamics. In fact, Betzel et al. (2012) who investigated EEG synchronization dynamics using another approach revealed three families of dynamic states for a broadband (4–30 Hz) network, whose edges were subdivided into three edge communities (i.e., a set of edges whose time courses are strongly correlated with one another).

As mentioned above, the duration of dynamic states determined by network similarity analyses, which is equal during oddball task and resting state, was longer than the ISI during the task. In other words, this dynamics was not directly related to stimulus impact and can hardly describe neural processes related to the stimuli. To answer the question whether there is another network dynamics that would be able to describe the network changes related to the task stimulation, we used the FNN classifier trained by a standard back-propagation algorithm. We found that the FFN classifier was able to differentiate between different ISIs with a total accuracy of 91.6%. This classification was independent of the task condition (attended or unattended) and the driving oscillation frequency. However, there were significant differences in *CA* between different GTA measures; *CC* and *E*_*loc*_ differentiated between different ISIs less accurately than other GTA measures. *CC* and *E*_*loc*_ are measures of network segregation indicating cliquishness of a typical neighborhood and efficiency of information transfer in the immediate neighborhood of nodes (Watts and Strogatz, 1998; Latora and Marchiori, 2001). Apparently, the local dynamics described by these two measures is more similar for different ISIs. We consider this result to be important, and worth further investigation.

Finally, some limitations of the present study need to be acknowledged. First, the present analyses considered only low frequency oscillations. However, a broader frequency range including high frequency oscillations (e.g., gamma) may provide additional information about network dynamics. Second, we used a sliding window approach to determine the coupling dynamics; probably some pointwise coupling measures may be more appropriate. Third, we used a phase-to-phase CFC; other types of CFC (e.g., phase-to-amplitude, phase-to-frequency, amplitude-to-amplitude, or amplitude-to-frequency; cf. Jirsa and Müller, 2013) may be (additionally) used for HFN construction providing additional information to frequency, phase, and/or amplitude interaction in brain dynamics. Finally, there are other different approaches to characterize and to differentiate brain state dynamics via hidden Markov models (Ou et al., 2015; Sourty et al., 2016; Vidaurre et al., 2016), dynamic Bayesian variable partition model (Smith et al., 2006; Zhang et al., 2014) or other models or algorithms (Dinov et al., 2016). It would be interesting to know whether there is a convergence in the determination of brain state dynamics when using different approaches.

We conclude that the NTD during rest and stimulus processing found using HFN approach reflects temporal and topological changes in the functional organization and reorganization of cortical networks and underlying neuronal cell assemblies.

## Author contributions

VM, TV, and UL designed the study, VM acquired and analyzed the data. VM, DP, TV, RS, VJ, and UL discussed the results and wrote the article. All authors read and approved the final version of the manuscript.

### Conflict of interest statement

The authors declare that the research was conducted in the absence of any commercial or financial relationships that could be construed as a potential conflict of interest.
